# Influence of the TGF-β Superfamily on Osteoclasts/Osteoblasts Balance in Physiological and Pathological Bone Conditions

**DOI:** 10.3390/ijms21207597

**Published:** 2020-10-14

**Authors:** Jessica Jann, Suzanne Gascon, Sophie Roux, Nathalie Faucheux

**Affiliations:** 1 Laboratory of Cell-Biomaterial Biohybrid Systems, Department of Chemical and Biotechnological Engineering, 2500 Boulevard Université, Université de Sherbrooke, Sherbrooke, QC J1K 2R1, Canada; Jessica.Jann@usherbrooke.ca (J.J.); Suzanne.Gascon@USherbrooke.ca (S.G.); 2 Department of Rhumatology, CHUS, 3001 12th Avenue N, Sherbrooke, QC J1H 5N4, Canada; Sophie.Roux@USherbrooke.ca; 3 Centre de Recherche du CHUS, 3001 12th Avenue N, Sherbrooke, QC J1H 5N4, Canada

**Keywords:** bone morphogenetic proteins, osteoclastogenesis, osteogenic differentiation, smad signaling pathway, RANKL

## Abstract

The balance between bone forming cells (osteoblasts/osteocytes) and bone resorbing cells (osteoclasts) plays a crucial role in tissue homeostasis and bone repair. Several hormones, cytokines, and growth factors—in particular the members of the TGF-β superfamily such as the bone morphogenetic proteins—not only regulate the proliferation, differentiation, and functioning of these cells, but also coordinate the communication between them to ensure an appropriate response. Therefore, this review focuses on TGF-β superfamily and its influence on bone formation and repair, through the regulation of osteoclastogenesis, osteogenic differentiation of stem cells, and osteoblasts/osteoclasts balance. After introducing the main types of bone cells, their differentiation and cooperation during bone remodeling and fracture healing processes are discussed. Then, the TGF-β superfamily, its signaling via canonical and non-canonical pathways, as well as its regulation by Wnt/Notch or microRNAs are described and discussed. Its important role in bone homeostasis, repair, or disease is also highlighted. Finally, the clinical therapeutic uses of members of the TGF-β superfamily and their associated complications are debated.

## 1. Introduction

Bone tissue plays several essential physiological roles within the human body, in particular mechanical functions such as protection, rigid support, and an anchoring site for soft organs (skeletal muscles) and metabolic functions [1,2]. Indeed, as the storage place of essential minerals (calcium and phosphorus), bone is a dynamic tissue in perpetual remodeling, alternating the phenomena of resorption and bone formation, which ensures the control of bone and phosphocalcic homeostasis of the human body [2,3]. There are two major families of bone cells with complementary activities—bone-forming cells (osteoblasts, osteocytes, and lining cells) and bone-resorbing cells (osteoclasts). The osteoblast/osteoclast balance is essential in bone homeostasis and its remodeling process, as well as in the repair of bone fractures. Any imbalance in their activity leads to diseases such as osteoporosis and Paget’s disease, which have strong consequences on the patient’s quality of life [4]. For example, osteoporosis not only increases the risk of bone fracture [5], but it also impairs bone’s inherent ability to self-renew, leading to non-unions [4,6,7].

Many cytokines, growth factors, hormones (PTH), and vitamins are involved in the phases of bone repair and remodeling [8,9,10,11]. The growth factors belonging to the TGF-β (transforming growth factor β) superfamily (particularly bone morphogenetic proteins (BMP) and TGF-β1) are known to act locally on bone formation, by stimulating the proliferation and chondrogenic/osteogenic differentiation of mesenchymal stem cells (MSCs) [12]. Thus, they constitute promising local therapeutic agents to promote bone repair. However, their roles on osteoclasts are still not well understood [13,14].

The TGF-β superfamily is an evolutionary conserved family of structurally related dimeric factors. They are secreted growth factors, which act as multifunctional regulatory peptides involved in a wide range of processes, including cell development, proliferation, and differentiation; wound healing; and carcinogenesis [15,16]. The TGF-β superfamily consists of several subfamilies, the TGF-β, Nodal, Activin subfamily and the BMP, growth and differentiation factor (GDF), anti-Müllerian hormone/Müllerian inhibiting substance (AMH/MIS) subfamilies.

In this review, the roles of bone-forming and bone-resorbing cells and their cooperation in healing and bone remodeling processes are presented. We will then introduce the role of the members of the TGF-β superfamily, their canonical/non-canonical signaling pathways and their respective regulations (Wnt/Notch, antagonist proteins, microRNAs) and discuss the complexity of their mechanisms that influence homeostasis and bone disease. Finally, the controversial clinical uses of members of the TGF-β superfamily in orthopedic surgery is debated.

## 2. Osteoblast/Osteoclast Balance in Bone Remodeling and Repair

### 2.1. Bone Forming Cells

#### 2.1.1. Osteogenic Differentiation

Osteoblasts develop from MSCs or osteoprogenitor cells. MSCs/progenitors can differentiate into chondrocytes, osteoblasts, or adipocytes, in response to specific growth factors and cytokines, such as BMPs and Wnt [17,18,19]. The source of osteoblast progenitors in vivo is still under debate. They can be found in bone marrow (MSCs accounting for 0.001 to 0.01 % nucleated cells) and periosteum [20,21]. Recently, new osteoprogenitors called transcortical perivascular cells (2–3% of Lin^−^cells from the digested cortical bone fraction) were identified [22].

The commitment of MSCs/progenitors to the osteoblast lineage depends on the activation of several transcription factors, such as the runt-related transcription factor 2 (Runx2) that acts upstream from Osterix (*Sp7* encoding for Osterix (Osx)) [23,24,25]. Runx2 is also involved in the proliferation of osteoprogenitor cells, by inducing the expression of the genes encoding fibroblast growth factor (FGF), FGF-2, and FGF-3 [26]. Both Osterix and Runx2 are required to induce the expression of genes encoding osteogenic markers [27]. In addition, the transcriptional activity of Runx2 and Osterix depends on their phosphorylation state at specific Ser residues [28,29].

In contrast, PPARγ (peroxisome proliferation-activated receptor γ) and CEBPα (CCAAT-enhancer binding protein α) are transcription factors that promote the adipogenic commitment of MSCs [30]. However, activation of Runx2 in MSCs appears to prevent their commitment into the adipocyte lineage [31]. The mechanisms based on Wnt and MAPK (Mitogen-activated protein kinase) pathways that control reciprocal expression of Runx2 and PPARγ and their phosphorylation state are essential in MSCs fate determination [32].

#### 2.1.2. Osteoblast and Osteocyte Functions

Osteoblasts that represent around 5% of the bone resident cells are located at the bone surface [33]. They are responsible for the organic matrix synthesis called osteoid and its mineralization. These cells mainly synthesize type I collagen (90% of osteoid), adhesion proteins (e.g., fibronectin, thrombospondin (TSP)), members of small integrin-binding ligand N-linked glycoprotein (SIBLING) family-like bone sialoprotein (BSP), and osteopontin, as well as proteoglycans (e.g., decorin, biglycan) [34,35,36]. The mineralization process, which leads to the nucleation and growth of hydroxyapatite microcrystals [Ca_10_(PO_4_)_6_(OH)_2_], is still under investigation (for review see [37]).

When mature osteoblasts are surrounded by secreted extracellular matrix, they undergo some morphologic changes characterized by a decreased volume, number of organelles, and star-shaped cell, to become osteocytes (for review on osteocytes see [38]). These cells, accounting for 90–95% of all resident bone cells, can survive several decades, depending on bone turnover rate, unlike osteoblasts (up to 5 months) and osteoclasts (few days) [39,40]. The osteocytes are now considered to be mechanosensory and endocrine cells that play a crucial role in bone homeostasis and remodeling, by regulating both osteoclast and osteoblast functions [38].

### 2.2. Bone Resorbing Cells

#### 2.2.1. Osteoclastogenesis

The multinucleated giant mature osteoclasts, accounting for 1% of all resident bone cells, are derived from myeloid precursors through the macrophage/dendritic cell lineage, following a multistep process called osteoclastogenesis. This process takes place in the bone marrow, adjacent to bone surfaces [33,41]. First, monocyte/macrophage precursor cells are committed into the osteoclast lineage. After a first phase of proliferation that is essential for differentiation to occur, the mononuclear osteoclastic precursors merge together, and gradually acquire the characteristics of multinucleated osteoclasts. The osteoclastic markers appear (tartrate-resistant acid phosphatase (TRAP), calcitonin receptor (CTR), α_v_β_3_ integrin), while the macrophagic markers disappear (nonspecific esterase (NSE), Mac-1). Then, they finally undergo maturation after adhesion to bone, in order to become polarized active osteoclasts that can form resorption lacunae [42].

**Figure 1:**
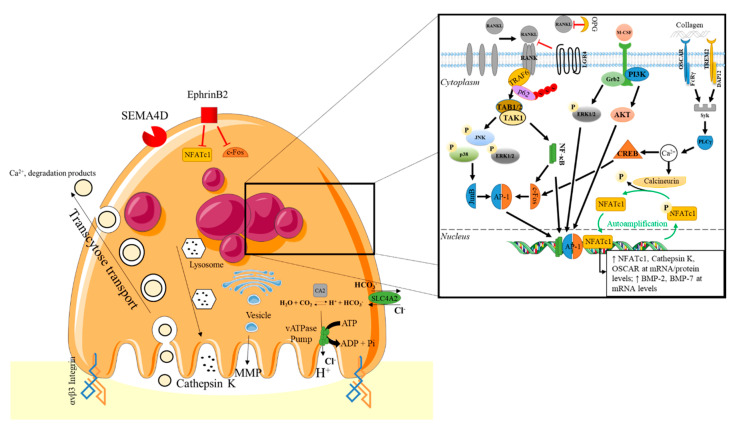
Osteoclast inducing bone resorption and its regulation by M-CSF, RANKL, and OSCAR/TREM2 signaling [55,56,57,58,59]. AP1: activator protein 1; CA2: carbonic anhydrase enzymes; CREB: cyclic AMP Response Element-binding protein; DAP12: DNAX associated protein 12kD size; ERK: extracellular signal-regulated kinase; Grb2: growth factor receptor bound protein 2; JNK: c-Jun amino (N)-terminal kinases; LGR4: Leucine rich repeat containing G-coupled receptor 4; M-CSF: macrophage- colony stimulating factor; NFATc1: nuclear factor of activated T cells; NF-κB: nuclear factor of κB; OPG: Osteoprotegerin; OSCAR: osteoclast-associated receptor; PI3K: Phosphoinositide 3-kinase; PLCγ: phospholipase Cγ; SLC4A2: Solute Carrier Family 4 Member 2; TAB1-2: TAK1-binding protein 1-2; TAK1: transforming growth factor β-activated kinase 1; TRAF: TNFR-associated factors; TREM2: Triggering receptor expressed on myeloid cells-2. The figure was created using Servier Medical Art. https://smart.servier.com.

Osteoclastogenesis mainly depends on two cytokines, the macrophage-colony stimulating factor (M-CSF) and the receptor activator of nuclear factor kappa beta ligand (RANKL) [43] (for review see [44]; Figure 1). M-CSF, also called colony stimulating factor 1 (CSF-1), is expressed by various cells including adipogenic mesenchymal stromal cells (adipocytic-primed leptin receptor positive cells), bone lining cells, osteoblasts, as well as microvascular endothelial cells [45,46,47]. M-CSF is recognized by the CSF-1 receptor c-Fms. Upon binding to its receptor, M-CSF activates the phosphoinositide 3-kinase (PI3K)/Akt and growth factor receptor bound protein 2 (Grb2)/extracellular signal-regulated kinase (ERK) pathways, leading to osteoclast precursor proliferation and survival [48].

RANKL—also called ODF (osteoclast differentiation factor), OPGL (osteoprotegerin ligand), or TRANCE (tumor necrosis factor-related activation-induced cytokine)—is expressed by osteogenic stromal cells, osteoblasts, proliferative chondrocytes, and lining cells [49,50]. Osteocytes are a major source of RANKL [51,52], and osteocyte-derived RANKL is essential for osteoclast formation [53]. RANKL can exist as a transmembrane protein or soluble form, after its cleavage by proteases [54].

RANKL binds to RANK a member of the tumor necrosis factor (TNF) receptor superfamily found on osteoclast precursors [60]. It was also recently found that the N-terminal extracellular domain of LGR4 (leucine rich repeat containing G-coupled receptor 4) compete with RANK to bind RANKL [61]. Upon RANKL binding to RANK, a homotrimeric transmembrane protein complex is formed, which induces the recruitment of the TNFR-associated factors (TRAFs), like TRAF6, leading to TAB1-2 ((TAK1-binding protein 1-2)/TAK1 (transforming growth factor β-activated kinase 1)) activation [60]. The p62 scaffolding protein, encoded by *SQSTM1*, is one of the functional links reported between RANKL and TRAF6-mediated signals [62]. Then, several intracellular pathways such as MAPK (p38, JNK, and ERK) or Akt are activated, leading to the stimulation of transcription factors, such as activator protein 1 (AP-1), nuclear factor of κB (NF-κB), Micropthalmia-associated transcription factor (MITF), c-Fos, or the master transcription regulator nuclear factor of activated T cells (NFATc1). These transcription factors are essential for the osteoclastogenesis and osteoclast maturation, by promoting the expression of genes encoding TRAP, v-ATPase subunit d2 (Atp6v0d2), osteoclast-associated receptor (OSCAR), β3 integrin subunits, and cathepsin K [63]. Indeed, specific receptors such as DAP12 (DNAX associated protein 12kD size) and FcRγ, as well as integrins (α_v_β_3_ and α_v_β_5_), play a crucial role in the osteoclastogenesis and osteoclast function [64,65,66]. For example, FcRγ and DAP12, with their respective associated receptors OSCAR and TREM2 (triggering receptor expressed on myeloid cells-2) are essential for NFATc1 activation via Syk/PLC/Ca^2+^ downstream signaling [64].

Several pro-inflammatory cytokines such as TNF-α and interleukins (IL) like IL-1, IL-7, IL-8, IL-11, and IL-15, can induce the osteoclastogenesis [67,68,69]. For example, TNF-α in vitro induced the formation of multinucleated cells’ ability to resorb bone in the presence of M-CSF [68].

TNF-α, IL-1β, IL-6 IL-7, and IL-15 can also induce the expression of M-CSF or RANKL [70,71]. Nevertheless, there are some discrepancies in the literature regarding the role played by some ILs, such as IL-6, during osteoclastogenesis [72,73,74]. For example, IL-6 (100 ng/mL) appears to suppress the osteoclast progenitor differentiation induced by M-CSF plus soluble RANKL (100 ng/mL) in vitro [73]. In contrast, the inhibition of IL-6 receptor by antibodies decreased the osteoclast formation by both mouse spleen cells treated with M-CSF (30 ng/mL, 24h alone before adding RANKL) and RANKL (50 ng/mL) in vitro and in TNFα–transgenic mice in vivo [75].

#### 2.2.2. Mature Osteoclast Functions

Osteoclasts are the only known cells that can resorb bone. This resorption depends on the ability of osteoclasts to interact with the bone matrix. At early stages of differentiation in vitro, osteoclasts form a primary specialized adhesion structure called podosome [76,77]. These podosomes are first organized into clusters associated with an actin cloud that evolve into a ring pattern [77]. At the periphery of mature osteoclasts, the podosome rings fuse together to finally form a structure called a belt or a sealing zone, when the cells adhere to the bone matrix [77,78]. Park et al. recently found that interaction between RACK1 and c-Src mediated by TRAF6 is required for osteoclast-mediated actin ring formation and cytoskeletal reorganization, during the process of bone resorption [79].

The sealing zone, characterized by the formation of the bone-apposed ruffled border membrane and the isolated resorption lacuna, segregates the resorptive microenvironment from the general extracellular space (Figure 1) [80,81]. The ruffled border membrane is involved in the transport into the resorption lacuna of proteolytic enzymes, as well as protons, via the vacuolar protons-transporting adenosine triphosphatase (v-ATPase) pump. The protons H^+^ are obtained by the activity of carbonic anhydrase enzymes (CA2) that catalyze the production of carbonic acid (H_2_CO_3_) from carbon dioxide and water [82]. The released protons allow the dissolution of the mineralized components of the bone matrix. The organic matrix made of type I collagen is then broken down by proteases such as Cathepsin K, which can also activate the matrix metalloproteases MMP-9, through cleavage [83,84]. Furthermore, it was recently shown that the deletion of both *Mmp9* and *Mmp14* genes in bone-marrow-derived myeloid cells altered the bone resorption activity of osteoclasts [85].

### 2.3. Osteoblasts/Osteoclasts Balance

#### 2.3.1. Bone Remodeling

Bone remodeling is a physiological dynamic and continuous process in which old bone is degraded and replaced to maintain its strength and mineral homeostasis. Osteoclasts and osteoblasts orchestrate the bone remodeling process via the formation of ‘basic multicellular unit’ (BMU) [81]. For example, the human adult skeleton has about 1–2 million active BMUs that function in an asynchronous manner to renew 3–10% of the bone tissue per year [38]. The bone remodeling process can be divided into six major phases [86]. The first one is the quiescence phase, followed by the second phase, called activation. The second phase is initiated by the activation of osteocytes induced by mechanotransduction or apoptosis of neighboring osteocytes, placed in a hypoxic environment, due to bone microcracks formation. The activated osteocytes in turn release several pro-inflammatory cytokines, such as TNF-α, which are known to attract osteoclast progenitors and promote their differentiation [68]. It was also proposed that osteocyte apoptosis directly promotes the osteoclastic bone resorption activity, but the soluble factors involved in this phenomenon were not identified. Indeed, osteoprotegerin (OPG), soluble decoy receptor that sequesters RANKL, was undetected [87]. However, another study found that there is a constant baseline bone remodeling, which is independent of the osteocyte apoptosis, when there are fewer than 45 apoptotic osteocytes/mm^2^ [88]. The third phase is the resorption, which implies that recruited osteoclast progenitors must undergo complete osteoclastogenesis, to become mature osteoclasts. The release of RANKL by osteocytes and osteoblasts is strongly involved in this phase. Mature osteoclasts degrade bone matrix to generate Howship’s resorption lacunae, by dissolving the mineral phase and degrading the organic matrix through specific collagenases (MMP) and proteases (as described in Section 2.2.2). The fourth phase is the reversal that is characterized by the removal of collagen fragments and debris by “osteomacs”, and the death of almost all osteoclasts through apoptosis [89]. During this phase, the recruitment of the osteoprogenitors begins, such as that of the bone lining cells, which are also major contributors of preosteoblasts in bone remodeling [49]. The fifth phase, the bone formation, is induced by the differentiation of recruited osteoprogenitors and the formation of mineralized bone matrix, by mature osteoblasts. The sixth phase, the terminal phase, includes the arrest of bone matrix synthesis through terminal differentiation of the embedded osteoblasts into osteocytes. The osteoblasts can also die by apoptosis (around 50–70%) or become bone lining cells. The osteocytes are involved in this arrest through the local release of sclerostin [90,91]. Indeed, the overexpression of *SOST* (gene encoding sclerostin) in transgenic mice reduces the bone mass [92]. Furthermore, the patients suffering from sclerosteosis and van Buchem disease (also known as hyperostosis corticalis generalisata), characterized by high bone mass, present a loss of the *SOST* gene function and *SOST* deletion on chromosome 17q (17q12–21 deletion), respectively [93,94].

Thus, the communication between osteoblasts/osteocytes and osteoclasts, play a crucial role during the bone remodeling process [95]. The osteoblasts/osteocytes can regulate the osteoclastogenesis by synthetizing RANKL or OPG (decoy receptor sequestering RANKL), which can promote or suppress osteoclastogenesis, respectively [96]. For example, under mechanical loading, the osteoblasts synthesize OPG via IL-6 stimulation, decreasing osteoclast formation [97]. In addition, the synthesis of sclerostin by osteocytes is decreased under mechanical stimulation, enabling bone formation [98]. Osteoblasts can produce semaphorins such as Sema3A, which interacts with neuropilin-1 present in the membrane of bone marrow-derived monocyte/macrophage precursors, to inhibit osteoclastogenesis. In contrast, osteoclasts, by expressing Sema4D through its binding to plexin-B1 receptors on osteoblast, inhibits bone formation [99,100]. Osteoclasts and osteoblasts can also interact together through their respective protein, ephrinB2 and EphB4. EphrinB2 favor osteogenic differentiation and osteoblast survival by limiting apoptosis, while it inhibits bone resorption by preventing c-Fos-NFATc1 signaling [101,102].

#### 2.3.2. Bone Fracture Healing

Bone healing involved intramembranous and endochondral processes. Intramembranous process occurs in fractures without any bone fragment displacement, which are also mechanically stable. The healing of larger bone fractures involves both endochondral bone formation and intramembranous healing [103].

Endochondral process occurs in three major phases—inflammation and hematoma formation, then bone repair (fibrocartilaginous and bony callus formation), and finally bone remodeling [104]. During the phase of inflammation and hematoma formation, the platelet–fibrin clot acts as a transitory scaffold that is able to recruit cells involved in the acute inflammation, via the presence of cytokines, such as IL-1 and IL-6, as well as chemoattractants like CXCL12 [105,106,107]. Interestingly, Burska et al. recently found an increase in IL-1β and IL-6, but not in TNF-α, during the early hematoma and inflammation phase in humans. Then, the levels of both IL-1 and IL-6 decrease while that of TNF-α increases [105]. The recruited neutrophils and M1 macrophages (until day 3) remove the damaged cells and tissue [108]. During the resolution of acute inflammation, macrophages evolve to M2 phenotype, and the MSCs are recruited by a gradient of cytokines and chemoattractants, such as CXCL12 and MCP-1 (also called CCL2) [8,105,108]. The bone repair phase is initiated by the formation of the fibrocartilaginous callus. The recruited MSCs differentiate into chondrocytes, which synthesize and secrete a cartilage matrix made of type II collagen and glycosaminoglycans [109]. Then, chondrocytes become hypertrophic and mineralize the cartilaginous matrix. After the death of hypertrophic chondrocytes through a process that might be independent of apoptosis, a transition from fibrocartilagenous callus to bony callus occurs [110]. It is promoted by angiogenesis (vascular endothelial growth factor, VEGF) and the differentiation of precursor cells into mature osteoblasts leading to bone formation and mineralization [9]. Finally, the last stage of bone healing is the bone remodeling, which involves both osteoclasts and osteoblasts, as described in Section 2.3.1.

Each phase of bone fracture repair and remodeling requires different hormones (PTH, 1,25-(OH)2D3), cytokines, growth factors, such as insulin like growth factor (IGF), FGF, and members of the TGF-β superfamily synthesized by bone cells [8,9,10,11]. Before describing the regulation of bone fracture healing phases by the TGF-β superfamily, this review first introduces the members of this superfamily, their signaling pathways, as well as crosstalk with Wnt and Notch signaling.

## 3. The TGF-β Superfamily

### 3.1. Members of the TGF-β Superfamily

To date, the TGF-β superfamily contains more than 30 members, including the TGF-β /Nodal/Activin (Inhibin) family, the BMP/growth differentiation factors (GDF) family, and the group of anti-Müllerian hormone/Müllerian inhibiting substance (AMH/MIS).

Members of the TGF-β superfamily are secreted growth factors, which act as multifunctional regulatory proteins in bone, being involved in a wide range of processes, including the proliferation, differentiation, and function of bone cells. They also coordinate the communication between osteoblasts and osteoclasts to ensure an appropriate response.

#### 3.1.1. TGF-β /Nodal/Activin Family

- TGF-β

TGF-βs were discovered by De Larco and Todaro [111]. Using the cell culture supernatant of mouse 3T3 fibroblasts transformed by a Moloney murine sarcoma virus, a family of growth-stimulating polypeptides called sarcoma growth factors (SGFs) was first identified and purified [111]. These SGFs not only stimulated the proliferation of the fibroblasts in a monolayer culture, but also acted as “effectors of fibroblastic cell transformation”, allowing them to grow in an anchorage-independent manner, in soft agar [111]. Further studies were then carried out to identify and purify TGF-βs from SGFs and other tissues [112,113,114].

There are three TGF-β isoforms in mammals, TGF-β1, TGF-β2, and TGF-β3, each encoded by genes located at different chromosomes (in human chromosome 19, 1, and 14, respectively) [115,116,117]. The TGF-β isoforms are synthesized as pre-pro-TGF-β monomers [118]. Each monomer contains an N-terminal signal peptide (SP, 29 amino acid residues), a pro-region called latency associated peptide (LAP, 249 amino acid residues) for proper folding of the growth factor, and a C-terminal mature growth factor domain (112 amino acid residues) [119]. After SP removal by cleavage, pro-TGF-β are dimerized via the formation of disulfide bonds. In the trans Golgi, the LAP dimers are then cleaved from the dimeric growth factor domains, by the endopeptidase furin, but remain non-covalently bonded to them. Thus, TGF-βs are usually secreted as latent complexes containing the dimeric growth-factor domains noncovalently bond with LAP dimers [118]. These complexes can also interact via the LAP dimers with other extracellular matrix components such as fibrillin, and latent TGF-β binding protein (LTBP), favoring the sequestration of the growth factor into the matrix for later activation [120]. In fact, these interactions might stabilize the latent TGF-β state due to the cross-armed conformation of the pro-TGF-β complexes, the growth factor remaining unable to interact with its Thr/Ser kinase receptors [121]. Indeed, Mi et al. hypothesized that cross-armed conformation of TGF-β family members corresponds to a latent state of the growth factor, while the open-armed conformation characterizes its mature active form [121]. Mature active TGF-β can be released from LAP and LTBP, through different latent TGF-β activators like proteases or membrane receptors. For example, α_v_β_6_ or α_v_β_8_ integrins that recognize the Arg-Gly-Asp motif in the pro-domains of TGF-β1 and TGF-β3, can exert a tensile force across the LTBP–LAP-TGF-β complexes, to release the mature form of the growth factor [120,122].

The sequence homology analyses revealed a high percentage of amino acid identity between the mature forms of the TGF-βs, which varied from around 71% (TGF-β1 and TGF-β2) to 80% (TGF-β3 and TGF-β2) [123,124]. However, several studies showed non-overlapping phenotypes of TGF-β1, TGF-β2, and TGF-β3 in knockout mice, suggesting that various functions are not compensated by other TGF-β isoform [125,126,127]. For example, using TGF-β1 null mutation in the homozygous state, Kulkarni et al. observed intrauterine death for around 65% of the embryos. The surviving mice that appear clinically normal at birth, develop uncontrolled inflammatory response in the heart and lungs, after 14 days, leading to their death within 2 weeks [125]. In contrast, TGF-β3-null embryos show a different phenotype from these TGF-β1 knockout mice with major deficiency in the palatal shelf fusion process [126]. Finally, TGF-β2 knockout mice possess aberrant skeletogenesis (skeletal induction and growth). In addition, while 66% of TGF-β2-deficient mice die shortly before or during birth because of multiple developmental defects, especially those affecting the heart, the surviving mice are cyanotic [127].

- Activin/Nodal

This review does not describe Nodal, despite its role in embryonic development and maintenance of stem cell pluripotency, because it is not expressed in adult non-neoplastic tissues [128] (for review see [129]). Activin was first discovered in the 1980s by Vale et al. as a dimeric polypeptide consisting of two inhibin βA -chains linked by disulfide bonds, which are able to induce the synthesis and release of the follicle-stimulating hormone FSH [130,131]. In mammals, 5 activins (activin A, B, AB, C, and E) were identified. These were characterized by the combination of inhibin subunits (βa, βb, βc, βe) that formed homodimers or heterodimers. For example, the activin A is composed of inhibin βa dimer, while the activin AB is made of inhibin βa and inhibin βb. The active mature form of activin is obtained after the cleavage of the secreted pro-activin through proteases like furin releasing the N-terminal prodomain [132,133]. Knockout mice for activin A appear healthy at birth, despite the lack of whiskers, but die within 1 day due to abnormal craniofacial development (defective palate) [134,135].

#### 3.1.2. BMP/GDF Family

Urist discovered the biomolecules responsible for the new bone formation in 1965, called BMP, after implantation of HCl (0,6 N)-decalcified bone matrix in the rectus abnominus of several animal models (mouse, rat, guinea pig, rabbit) and rabbit quadriceps [136]. Currently 20 BMPs/GDFs are identified. Several classifications were proposed to regroup the members of the BMP/GDF family. For example, these were classified into seven subgroups based on the sequence residue homologies in their carboxy-terminal mature growth factor domain [137,138,139,140]. BMP-2 and BMP-4 with around 92% amino acid identities are members of the Drosophila decapentaplegic (dpp) subgroup (subgroup I). BMP-5, BMP-6, -BMP-7, and BMP-8 are members of the Drosophila 60A subgroup (subgroup II). These BMPs share less than 65% residues identities with BMP-2 [139]. BMP-9 (GDF 2) and BMP-10 with around 65% amino acid identities are members of the subgroup III. The other subgroups are: (IV) GDF5 (BMP-14), GDF 6 (BMP-13), and GDF 7 (BMP-12); (V) Myostatin (GDF8) and GDF 11; (VI) GDF 1 and GDF3; and (VII) GDF 10 and BMP-3 [137,138,139,140]. A classification of 14 BMPs into three subgroups was recently proposed [141]. This new classification was established based on the clustering analyses of 519 genes transcriptomic profiles (e.g., genes encoding Ser/Thr kinases, Noggin, Smad6, Smad7, Id, parathyroid hormone receptor 1, Wnt) in multipotent murine C3H10T1/2 stem cells transduced by adenovirus expressing BMPs. BMP-2, BMP-4, BMP-6, BMP-7, and BMP-9, which are well-known to induce multilineage differentiation of mesenchymal stromal cells, are members of the first subgroup. BMP-5, BMP-11, BMP-12, BMP-13, BMP-14, and BMP-15, which are involved in the repair of tendon and ligament injuries, are members of the second subgroup [141]. Interestingly, the third subgroup contains BMPs with various functions, such as BMP-3, BMP-8, and BMP-10. Indeed, BMP-3 is known as a negative regulator of bone density and bone formation [142], while BMP-8 and BMP-10 are involved in postnatal spermatogenesis and cardiac development, respectively [143,144].

As for TGF-βs, BMPs are synthesized as pre-pro-BMPs. For example, the pre-pro-BMP-9 contains a SP of 22 residues, a pro-domain of 297 residues and a 110 residues mature growth factor domain [145]. After SP removal, the pro-BMPs form dimers that are then cleaved by subtilisin-related pro-protein convertases (furin), favoring the formation of complexes through noncovalent association between the pro-domain fragments and the growth factor domain [145,146]. After secretion, the pro-BMP complexes can interact with the extracellular matrix to get a cross-armed conformation that induces the latency of the growth factor [147]. However, unlike pro-TGF-β1, some pro-BMP complexes such as pro-BMP-7 and pro-BMP-9 can also adopt an open-armed conformation after secretion. This conformation allows their binding to Ser/Thr kinase receptors and signal transduction, despite the presence of non-covalent interactions with the pro-domain fragments [121,148]. For example, using human pulmonary artery endothelial cells, Salmon et al. recently showed that pro-BMP-9 complexes and BMP-9 induce the same expression of the gene encoding the inhibitor of DNA binding protein 1 (ID1), suggesting a similar signal transduction efficiency [149].

Among the members of the BMPs/GDFs family, BMP-2, BMP-4, BMP-5, BMP-6, BMP-7, and BMP-9 are well-known to induce the differentiation of osteoprogenitor cells into osteoblasts [150,151,152,153,154]. However, the use of knockout mice revealed that some BMPs are not only involved in skeletogenesis, but also induce defects in several organs, such as heart, kidney, and lungs [155]. For example, most of the homozygous null *Bmp4* mutants die in early gastrulation, but the surviving embryos display a lack of allantois as well as primordial germ cells, both derived from precursors in the proximal epiblast [156,157]. In the same way, BMP-7-deficient mice die shortly after birth and not only have skeletal abnormalities in discrete areas such as rib cage, skull, and the hind limbs, but also eye and kidney defects [158].

### 3.2. TGF-β Superfamily Signaling Pathways and Their Regulation

#### 3.2.1. The Canonical Pathways Used by Members of TGF-β Superfamily

**Figure 2:**
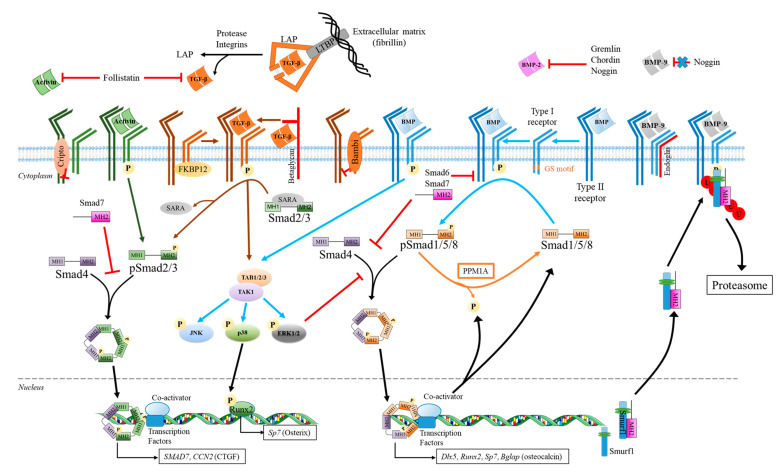
The TGF-β superfamily canonical and non-canonical pathways and their regulation for controlling the expression of targeted genes in osteoprogenitors and bone forming cells [120,133,159,216,217,218,219]. BAMBI: BMP and activin membrane-bound protein; FKBP12: FK506 binding protein of 12 kDa; LAP: latency associated peptide; LTBP: Latent TGF-β binding protein; PPM1A: protein phosphatase magnesium-dependent 1A; and SARA: Smad anchor for receptor activation protein. The figure was created using Servier Medical Art. https://smart.servier.com).

Members of the TGF-β superfamily act on cells by binding with different affinity to Type I and Type II Ser/Thr kinase receptors, leading to the activation of the canonical small mothers against decapentaplegic (Smad) or mitogen-activated protein kinase (MAPK) signaling pathways [159]. The Smad2/3 is activated by TGF-β/Nodal/Activin family and members of the BMP/GDF subgroups V, VI, and VII (GDF8/GDF11; GDF1/GDF-3; and BMP-3/GDF-10), while Smad1/5/8 (also recently called Smad1/5/9) is initiated by BMPs of subgroups I to IV (Figure 2) [160,161,162,163]. However, in rare situations, activin and TGF-β can also activate the Smad1/5/8 signaling [164,165]. Only a limited number of Type I and Type II Ser/Thr kinase receptors were identified in humans—seven type I (activin receptor-like kinases 1 to 7, ALK1-7) and five type II receptors (type II BMP receptor (BMPRII), type II activin receptor (ActRIIA), type IIB activin receptor (ActRIIB), TGF-βRII (TβRII), and anti-Mullerian hormone receptor type II (AMHRII)) [166,167,168]. These type I or type II receptors are characterized by a ligand-binding extracellular domain at their N terminal extremity, a single pass transmembrane part, and an intracellular domain at their C-terminal extremity, containing the Ser/Thr kinase activity [169,170].

Our research team detected ALK1, ALK3, ALK6, and BMPRII receptors at both mRNA and protein levels in multinucleated cells (Table 1). Using double-immunofluorescence staining, we confirmed that CTR and RANK positive cells (osteoclasts) express ALK1 and BMPRII [171]. However, Kaneko et al. found that unlike BMPRIB (ALK6), both BMPRIA (ALK3) and BMPRII were expressed in isolated rabbit mature osteoclasts [172].

The signal transduction induced by the members of the TGF-β superfamily can also depend on a third co-receptor. It does not possess any Ser/Thr kinase activity, but is able to control the ligand availability or increase the affinity between the ligand and its receptors, thus, controlling the signaling kinetics and intensity (for review see [203]). For example, the betaglycan, also known as TβR-III can interact not only with the three TGF-β isoforms, but also with BMP-2, BMP-4, and BMP-7 [204,205,206]. Another type III receptor called endoglin (ENG) appears to have affinity to TGF-β1 and TGF-β3 but not to TGF-β2, despite sequence homologies with betaglycan [207]. However, these observations remain controversial since Castonguay et al., using cell-based assays and surface plasmon resonance, found that ENG fails to bind TGF-β1 and TGF-β3, with or without type II receptor TβRII [208]. Nevertheless, ENG recently gained a lot of interest because of its involvement in endothelial cells response to members of the third BMP/GDF subgroup (BMP-9 and BMP-10) [209].

Six Smad proteins are known to transduce the signals of the TGF-β members from the cell surface to the nucleus (Smad2/3, Smad1/5/(8 or 9), and Smad4). These are transcription factors that contain two highly conserved domains—the Mad homology 1 (MH1) domain at their N-terminus and the Mad homology 2 (MH2) domain at their C terminus, which are connected through a poor conserved linker region. The MH1 and MH2 domains play a crucial role in DNA recognition/binding and Ser/Thr receptor interaction, respectively [210,211]. The linker region, rich in Pro and Ser/Thr residues, is “structurally flexible” and possesses several phosphorylation sites that control the ability of the Smad proteins to transduce the signal into the nucleus [212,213,214,215].

- Smad 2/3 Pathway

The activation of the canonical Smad2/3 pathway is initiated by the recognition of the dimeric ligands (members of the TGF-β /Nodal/Activin family and BMP/GDF subgroups V, VI, and VII) by a Type II receptor homodimer [220]. For example, all TGF-β isoforms can specifically interact with the TβRII receptors. However, while TGF-β1 and TGF-β3 bind TβRII with a high affinity (estimated K_D_ ∼200 pM and ∼500 pM, respectively), TGF-β2 binds TβRII with a low affinity (estimated K_D_ > 10 nM) [221].

The ligand-Type II receptor bindings induce a conformation change of the receptors making high affinity binding sites for Type I receptors accessible. Three type I receptors, ActRIb/ALK4, TβRI/ALK5, and ALK7, can initiate the TGF-β/Nodal/Activin signaling [162]. However, TβRII transduce the TGF-β signal exclusively by forming heterooligomers with ALK5. In the same way, ALK4 is described as the main type I receptor for activin A [222,223]. Upon their recruitment, an allosteric conformation change of the Type I receptors occurs. It allows the release of the FK506 binding protein of 12 kDa (FKBP12) from Type I receptors. These type I receptors are then activated via the phosphorylation of their Gly/Ser rich motif (GS motif), located adjacent to their kinase domain by type II receptors [224]. Upon phosphorylation, they have a higher affinity for the MH2 domains of Smad2/3 proteins, thus promoting the Type I receptors-Smad2/3 interaction [219].

It was suggested that the specific interaction between the Type I receptors and Smad2/3 proteins are mediated via their L45 loop in the kinase domain and L3 loop in MH2 domain, respectively. However, the amino acid sequence of the L45 loop (a loop in the N-lobe of the receptor) is identical between ALK4, ALK5, and ALK7 [225,226]. The subcellular localization and presentation of Smad2/3 to type I receptors appears also to involve several proteins, such as the Smad anchor for receptor activation protein (SARA) located in early endosomes [227,228].

The type I receptors then phosphorylate the Smad2/3 proteins at 2 Ser residues (*) in the SS*XS* motif, on their MH2 domain. Phosphorylated Smad2/3 also called the receptor-regulated Smad proteins (R-Smad) can then be dissociated from the receptors and interact with the L3 loop on the MH2 domains of Smad 4 (also called Co-Smad) to form heterotrimeric complexes. In fact, Tsukazaki et al. found that phosphorylation of Smad2 induces its dissociation from SARA but favors Smad2/Smad4 interaction [227]. These R-Smad/Co-Smad complexes are translocated to the nucleus, where they interact with specific DNA sequence (Smad-binding element) via the Smad3 MH1 domains and the cooperation of other transcription factors (TFE3), to induce the transcription of specific genes (*SMAD7*) [229,230]. The ability of Smad2 to interact with DNA requires an open conformation of its E3 insert on the MH1 domain [231]. After the gene transcription, the nuclear Smad2/3-Smad 4 complexes can be dephosphorylated, dissociated from DNA, and recycled. The principal Smads in the TGF-β/Activin/Nodal pathways lead to target genes different from those controlled by the Smads in the BMP pathways [16].

Several studies observed the activation of the Smad canonical pathway induced by TGF-β1 in osteoclast precursors and mature osteoclasts (Table 1). For example, Gratchev et al. showed that TGF-β1 (10 ng/mL) induces the activation of the Smad2/3 signaling pathways after only 10 min of stimulation [176]. Furthermore, this stimulation is 10 times greater in mature human macrophages than in non-mature ones [176]. Activation of this signaling pathway mediates the expression of other factors that play a key role in cell differentiation. Ota et al. showed that the expression of Wnt10b factor by TGF-β1 (2 ng/mL) is dependent on the activation of Smad2/3 in osteoclasts but independent of other signaling pathways (Akt or MAPK) [177].

- Smad1/5/8 Pathway

The activation of the canonical Smad1/5/8 pathway is primarily initiated by the BMP homodimers (subgroups BMP subgroups I to IV) or heterodimers binding to Ser/Thr kinase receptors by their wrist epitopes (type I receptor interaction), and knuckle epitopes (Type II receptor interaction) [140,162]. In fact, when BMP dimer binding induced the receptor oligomerization, the Smad1/5/8 pathway is favored. In contrast, BMP dimer interaction with preassembled receptor complexes induce the MAPK pathway activation [232,233]. BMP members of the dpp, 60A, and third (BMP-9/BMP-10) subgroups bind several type II receptors (BMPRII, ActRIIA, and ActRIIB) with different affinities [234]. For example, BMP-2 has a lower affinity for ActRIIA than BMP-7 (Kd = 24 nM for BMP-2; Kd = 8 nM for BMP-7). The type I receptors ALK1, ALK2, ALK3 (BMPRIa), and ALK6 (BMPRIb) can also trigger the BMP signaling. For example, BMP-9 binds to ALK1 with a high affinity, but it can also transduce its signal through ALK2 [140,235,236]. BMP-2 and BMP-4 (dpp subgroups) are mainly recognized by ALK3 and ALK6 [234,236,237]. Interestingly, Salmon et al. recently confirmed the findings of Mi et al. showing that pro-BMP-9 complexes can also bind to ALK1 via a partial but not entire displacement of their pro-domain fragments (α5-helix) [121,149]. Using surface plasmon resonance analyses, they found that the K_D_ value of pro-BMP-9: ALK1-Fc complex (around 61 pM) is quite similar to that obtained with BMP-9 (around 48 pM) [149]. The pro-BMP-9 complexes can also selectively bind to type II Ser/Thr kinase receptors with different EC_50_ as compared to mature BMP-9. These complexes interact better with ActRIIB than BMPRII (EC_50_ for Fc-fused type II receptors of 0.02 nM and 1.6 nM, respectively), while BMP-9 similarly binds both receptors (EC_50_ for Fc-fused type II receptors of 0.04 nM) [121].

Upon BMP binding, the constitutively active Ser/Thr kinase type II receptors phosphorylate the type I receptors at their GS motif. The activated type I receptors in turn phosphorylate, Smad 1/5/8 on the SSXS motif, which can then interact with Smad 4 to form complexes [238]. These complexes translocate into the nucleus to regulate with other transcription factors, such as Runx2 and Osterix the expression of genes such as *BGlap1* encoding osteocalcin [239].

Few studies analyzed the signaling pathway induced by BMPs in osteoclasts (Table 1) [171,187]. Our research team found that rhBMP-9 at 150 ng/mL induces the Smad1/5/8 phosphorylation at 15 min in human osteoclasts. The Smad1/5/8 that remain phosphorylated within 2 h were translocated into the nucleus. In contrast as expected, the Smad2 phosphorylation levels following rhBMP-9 stimulation are faint, compared to TGF-β (10 ng/mL) [171]. On the other hand, Broege et al. showed that BMP-2 induces the activation of canonical (Smad) and non-canonical (MAPK) signaling pathways differently, depending on the stage of differentiation of bone marrow macrophages into osteoclasts [187]. BMP-2 at 30 ng/mL induces the activation of MAPK pathways (p38), at an early stage in pre-fusion osteoclasts (day 1 of differentiation), whereas Smad1/5/8 are phosphorylated during the fusion of osteoclast precursors (day 2–3 of differentiation) [187].

- Regulation Mechanisms of the Canonical Smad Pathways

**Figure 3:**
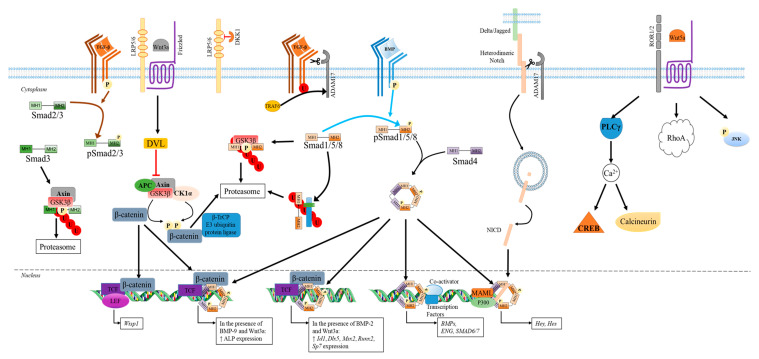
The effect of Wnt and Notch pathways on TGF-β superfamily signaling to control the expression of targeted genes in osteoprogenitors and bone-forming cells [216,217,274,275,276,277]. APC: adenomatous polyposis coli; β-TrCP: β-transducin repeat-containing protein; CKIα: Casein kinase Iα; Dkk1: Dickkopf1; DVL: Disheveled; ENG: Endoglin; GSK3 β: Glycogen synthase kinase-3 β; LEF: Lymphoid enhancer-binding factor; LRP5/6: low-density-lipoprotein-related protein 5/6; NICD: Notch intracellular domain; TCF: T cell factor; and U: ubiquitination. The figure was created using Servier Medical Art. https://smart.servier.com.

The canonical pathways activated by members of the TGF-β family can be inhibited by several mechanisms (Figure 3) [203]. The signal transduction induced by the members of the TGF-β superfamily can be regulated by the internalization of the cell-surface receptors, through clathrin-dependent mechanisms or cholesterol enriched caveola [233]. Inactive membrane receptor lacking the intracellular Ser/Thr kinase domain such as BAMBI (decoy-receptor BMP and activin membrane-bound protein) can also inhibit TGF-β, activing, and BMP signaling. BAMBI appears to act through interaction with receptors rather than TGF-β ligands, as shown by Onichtchouk et al., using a receptor affinity-labeling experiment with radiolabeled [^125^I] BMP-2 or [^125^I] TGF-β1 [240]. Interestingly, they also found that BAMBI can interact with all type I receptors except ALK2, and with TβRII and ActRII type II receptors [240]. In the same way, activin-βA and –βB that can signal through ALK4 and ActRIIA or ActRIIB are inhibited by the receptor Cripto-1 [241].

Another mechanism, preventing the signaling pathways of the TGF-β superfamily, is the use of antagonist proteins such as the Dan family (Gremlin), the Spemann organizer signal molecules (Noggin, Chordin) and follistatin [92,159,203]. These antagonist proteins are secreted into the extracellular space and selectively bind to certain members of the TGF-β superfamily, blocking the activation of their receptor and inhibiting the intracellular signaling [159,203]. The binding of Noggin and Chordin to BMP-2, BMP-4, and with lower affinity to BMP-7, prevents the recognition and interaction with their type I and type II receptors [167,242,243]. On the other hand, follistatin and follistatin-like proteins are the only secreted antagonists acting on activins, TGF-βs, and GDF8/myostatin [244,245].

Other regulatory mechanisms act directly in the cytoplasm. The deactivation of R-Smad can be obtained via their dephosphorylation by phosphatases, such as the protein phosphatase magnesium-dependent 1A (PPM1A). The canonical Smad pathway can also be blocked by intracellular molecules like Smad 6/7, also called I-Smad (Inhibitory Smad) [246]. Unlike R-Smad and Co-Smad, I-Smad contains only one conservative MH2 domain [214]. The MH2 domains of I-Smad, particularly the L3 loop, are essential for their association with activated type I receptors [247]. Smad6 primarily interferes with the signal transduction of BMPs, through ALK3 and ALK6 [248]. For example, the binding of Smad6 on ALK3 occurs exclusively through a motif of the MH2 domain, called the basic groove, comprising the L3 loop of the MH2 domain and α-helix 1 [249]. Smad7 uses two distinct structural motifs (the basic groove and the three-finger structure) to inhibit Smad signaling induced by TGF-β and BMPs [247,250]. The basic groove of Smad-7 interacts with the ALK5 receptor [249], while both three-finger-shaped structure and basic groove, are involved in interaction with ALK2, ALK3, and ALK4 receptors [247]. Interestingly, I-Smad can cooperate with other proteins to inhibit intracellular signaling by acting on activated type I receptors. For example, they can act with the E3 ubiquitin ligase Smurf (Smad ubiquitin regulatory factor), to favor the proteasome degradation of both TGF-β and BMP receptors upon their ubiquitination [251]. For example, BAMBI can act synergistically with Smad7 through a ternary complex with ALK5, to block the association of R-Smad (Smad3) with receptors, and their activation [203,252]. Furthermore, it was also suggested that Smad8/9 that displays a lower transcriptional activity than Smad1/5 can act as an inhibitor of BMP signaling [253,254].

It was recently shown that microRNAs (miRNAs) can play a strong role in the regulation of the signal transduction induced by the members of the TGF-β superfamily. MicroRNAs, which possess 18–25 nucleotides, are small noncoding RNA molecules that can inhibit the translation of targeted mRNAs or induce their degradation (for review see [255]). Both miR-422a and miR-153 inhibit the post transcriptional expression of the gene encoding TGF-β2 in osteosarcoma cells [256,257,258]. MicroRNAs such as members of the miR-30 family (miR-30a, -30b, -30c, -30d) can also downregulate the amount of Smad1 and Runx2, when introduced in MC3T3-E1 preosteoblasts treated by 200 ng/mL BMP-2, thus, preventing osteogenesis [259]. Interestingly, among the six members of the miR-30 family (miR-30a, -30b, -30c, -30d, -30e, and miR-384–5p), only the expression of miR-30a, -30b, -30c, and -30d is downregulated in murine MC3T3-E1 preosteoblasts treated by 200 ng/mL BMP-2, after incubation for 8h [259]. Li et al. also found that the introduction of miR-135 and miR-133 into MC3T3-E1 preosteoblasts, downregulates the expression of Smad5 and Runx2, respectively, and reduces the expression of markers of osteoblast differentiation (Alkaline phosphatase, ALP) [260]. In contrast, some other miRNA can promote osteogenesis by upregulating the expression of BMP and transcription factors or preventing the expression of their BMP pathway inhibitors [255,261]. The overexpression of miR-20A in human MSCs isolated from bone marrow, promotes their osteogenic differentiation. It also induces an increase in BMP-2/BMP-4 and Runx2 at both mRNA and protein levels. In addition, miR-20A downregulates the expression of the membrane receptor BAMBI [261].

#### 3.2.2. Non-Canonical Pathways Used by Members of TGF-β Superfamily

The members of the TGF-β superfamily through binding to their preformed type I and type II receptors can first activate XIAP, then TAK1 and TAB1, which in turn initiates the p38, ERK, and JNK (c-Jun amino (N)-terminal kinases) MAPK cascades [262,263,264]. For example, Li et al. found that the phosphorylation of ERK1/2 is decreased in the mouse spleen macrophage through BMP-9 treatment [265] (Table 1). In contrast, our research team showed that BMP-9 at 150 ng/mL induces an increase in the amount of phosphorylated ERK1/2, but not p38 in human osteoclast, after 5 min [171]. Moreover, Broege et al. showed that phosphorylation of p38 in murine pre-fusion osteoclasts is increased, following treatment during 15 min with BMP-2 (30 ng/mL) [187] (Table 1).

MAPK cascades can favor or prevent osteogenic differentiation. For example, MAPKs promote osteoprogenitor differentiation by upregulating the expression of Runx2 and Osterix [266,267]. MAPKs such as p38 and ERK1/2 can phosphorylate osteogenic transcription factors, especially Dlx5, Runx2 and Osterix, thus, promoting their activity [28,268,269,270]. In contrast, JNK1, by phosphorylating Runx2 at Ser104, reduces its transcriptional activity [271]. Furthermore, the MAPK pathway can also antagonize the BMP canonical Smad cascade by phosphorylating the linker region of Smad1, which inhibits Smad1 activity and might prevent its nuclear localization [215,272].

To summarize, the description of the signal transduction induced by the members of the TGF-β superfamily can appear simple—hetero-oligomerization of limited number of Type I and Type II receptors leading to 2 canonical Smad pathways activation. However, it must be kept in mind that the ligand pro-domains, ligand heterodimerization, binding receptor affinities, structure of both ligand-receptor complexes, with or without third co-receptors, and R-Smad/Co-Smad complexes also have strong effects, which are still under investigation (for review see [203,273]). Furthermore, other signaling pathways such as the Wnt and Notch cascades, are also able to regulate the signal transduction induced by the members of the TGF-β superfamily.

#### 3.2.3. Other Regulators of the TGF-β Superfamily

- Wnt Signaling Pathways

The Wnt proteins were discovered in 1980s due to the work of Nusse and Varmus who identified the *int-1* proto-oncogene in virus-induced C3H mouse mammary tumor model [278]. Indeed, the name Wnt is derived from a combination between the Drosophila segment polarity gene called *wingless* and its vertebrate homolog, *integrated* (*int-1*) [279]. To date, 19 genes encoding Wnt proteins are identified in mammals. The members of the Wnt family can transduce their signals inside the cells through 3 major pathways—one β-catenin dependent called the canonical Wnt pathway and two other β-catenin independent pathways, known as planar cell polarity (PCP) and Wnt/Ca^2+^ pathways, which are considered to be non-canonical [280].

The 3 major Wnt pathways act on cells through a family of seven-pass transmembrane Frizzled (Fz) receptors, which interact with Wnt, via their extracellular N-terminal cysteine-rich domain (CRD) (for review see [281] and [282]).

- Canonical Wnt pathways

When the canonical Wnt pathway remains inactive, β-catenin forms a destruction complex with the adenomatous polyposis coli (APC), Axin, casein kinase I (CKI), and Glycogen synthase kinase-3 β (GSK3 β). Then, β-catenin is phosphorylated by these kinases (CKI then GSK3) leading to its ubiquitination by β-transducin repeat-containing protein (β-TrCP), an E3 ubiquitin ligase [283,284]. Upon ubiquitination, β-catenin is transported to the proteasome for degradation, blocking any signal transduction inside the nucleus.

The canonical Wnt pathway is initiated by the binding of Wnt, such as Wnt1 and Wnt3A, to Fz receptor and a single-span transmembrane co-receptor called low-density-lipoprotein-related protein 5/6 (LRP5/6) (Figure 3). The co-receptors LRP5/6 can interact with Disheveled (DVL), which then binds to Axin. Axin prevents the phosphorylation of β-catenin, allowing its accumulation in the cytosol. Then, β-catenin can be translocated to the nucleus, where it activates TCF (T cell factor) and LEF (lymphoid enhancer-binding factor), to induce the expression of specific genes such as *Wisp-1*. The Wnt pathways can be antagonized by several proteins such as the Fz-related proteins (sFRPs), which are able to bind Wnt, preventing their recognition by the receptors. Other antagonists such as the members of the Dickkopf (Dkk 1, -2 and -4) interact with LRP5/6, inhibiting the canonical Wnt pathway [285].

The canonical Wnt pathway through the β-catenin is involved in the skeletal development, as well as the fracture healing process [286,287]. For example, Wnt can direct the fate of mesenchymal stromal cells and favors their osteogenic differentiation by upregulating the genes encoding for the transcription factor Runx2 and Osterix, while it limits their adipogenic differentiation by preventing the expression of the genes encoding CCAAT/enhancer-binding protein alpha and PPAR-γ [288]. In addition, β-catenin and TCF-1 can indirectly inhibit osteoclastogenesis, by favoring the expression of the gene encoding OPG in osteoblasts [289].

The Wnt pathway activation states are, therefore, able to regulate the signaling of TGF-β superfamily members and vice-versa [217,290,291]. For example, Guo et al. showed that unlike Smad2, the availability of Smad3 for type I receptor activation can be controlled by Axin and GSK3β. Indeed, Smad3 forms a destruction complex with Axin and GSK3β, independent of the β-catenin, allowing its phosphorylation at Thr66 by the kinase, its subsequent ubiquitination, and proteasome-dependent degradation. Furthermore, Axin depletion enhances Smad3 activation by TGF-β [292]. Fuentealba et al. also observed that Smad1 phosphorylation at its linker region by GSK3 leading to its polyubiquitination, is dependent on ERK prephosphorylation [293]. The activation of the Wnt pathways by Wnt3a stabilizes Smad1 by preventing its phosphorylation by GSK3 [293].

Some Wnt ligands can also promote a shift of the TGF-β signaling pathway from Smad2/3 towards Smad1/5/8. Using murine P2 chondrocytes, Van den Bosch et al. found that a Wnt3a (300 ng/mL) pretreatment is enough to decrease the amount of phosphorylated Smad2/3 induced by TGF-ß1 (5 ng/mL) for 30 min. In contrast, it increases the amount of phosphorylated Smad1/5/8, signaling involved in chondrocyte hypertrophy. Similar results were obtained with human G6 chondrocytes, and the effect on the shift in TGF-β-induced Smad phosphorylation is even stronger when Wnt3a is combined with WISP [164]. The addition of a specific inhibitor of the canonical Wnt pathway (Dkk-1) in vitro, as well as the use of Wnt8a in vivo, confirmed that this shift in TGF-β-induced Smad phosphorylation depends on the canonical Wnt pathway [164].

Several studies showed that the osteoblastic differentiation of osteoprogenitor cells can also be enhanced by some BMP and Wnt combination [294,295,296]. For example, murine C2C12 cells treated for 2 h by BMP-2 (2 nM) and Wnt3a (100 ng/mL) contained more mRNA encoding osteogenic markers Dlx5, Msx2, and Runx2 than those treated by BMP-2 or Wnt3a alone. These results were confirmed using primary mesenchymal stromal cells extracted from the bone marrow and cultured for 4 days in an osteogenic medium containing both BMP-2 and Wnt3a. The expression of genes encoding Id1, Dlx5, Msx2, Runx2, and Osterix, is synergistically increased by the cytokine combination. This synergistic effect is allowed by the formation of a cooperative Smad/TCF4/β-catenin transcriptional complex [295]. In the same way, using murine multipotent C3H10T1/2 cells infected by adenovirus (Ad) expressing BMP-9 or Wnt3a, Tang et al. found that Wnt3a enhances the BMP-9-induced ALP activity in a β-catenin dependent manner. The use of AdBMP-9 also appears to favor the expression of the late osteoblastic differentiation marker osteocalcin, through the formation of a Runx2/β-catenin/TCF transcriptional complex. The ectopic bone formation induced by the implantation of C3H10T1/2 cells transduced with AdBMP-9 in the flanks of athymic nude mice for 5 weeks, is also inhibited by β-catenin knockdown [294].

- Non-canonical Wnt signaling pathways

The PCP pathway implies the binding of Wnt such as Wnt5A to Fz or ROR/PTK7 co-receptors, to activate JNK and members of the small Rho GTPase family like RhoA and Rac1 [297]. The signal is transduced to the nucleus, activating the expression of targeted genes like *XPAPC (Xenopus paraxial protocadherin)* via some transcription factors like ATF2 [298]. It was shown that bone–marrow-derived macrophages (BMMs) secrete Wnt5a that can bind to their Ror2 receptors to promote RANKL expression, leading to their differentiation into mature osteoclasts. In addition, Wnt5a-Ror2 binding on mature osteoclasts stimulates RhoA involved in the actin ring formation in osteoclasts. It also promotes the activity of the C-Src/ Rho effector kinase (Pkn3) complex, increasing osteoclast bone-resorption activity [299]. Using osteoclast specific Ror2 conditional knockout mice, Uehara et al. observed an increase in bone mass due to altered actin ring formation and bone resorption [300].

The Wnt/Ca^2+^ pathway involves Fz-mediated phospholipase C (PLC) activation via heterotrimeric G proteins. PLC in turn catalyzes the diacylglycerol (DAG) and inositol-1,4,5-trisphosphate (IP3) production [301]. IP3 induces the Ca^2+^ release from intracellular endoplasmic reticulum to stimulate effectors such as calmodulin-dependent kinase II (CAMKII) and protein kinase C (PKC), which can activate, for example, the transcription factors NFκB and CREB (cyclic AMP response element-binding protein). Ca^2+^ and calcineurin can also activate the NFAT [302,303].

- Notch Signaling Pathways

Notch are cell-surface receptors (Notch 1–4 in mammals) that recognize Delta-like (DLL1, 3, and 4 in mammals) and Jagged (JAG1, 2 in mammals) single-pass transmembrane ligands on neighboring cells. The ligand-Notch receptor binding induces the intracellular cleavage of Notch by the TNFα-converting enzyme (TACE) or ADAM17 and γ-secretase complex. ADAM17 belongs to the ADAM (a disintegrin and metalloproteinase) family of proteins, which are transmembrane metalloproteinases, possessing a catalytic extracellular domain, and are involved in ectodomain shedding of various cell surface proteins, including growth factors, cytokines, receptors, and adhesion molecules [304]. The TβR1 receptor (ALK5) was previously shown to be a substrate of ADAM17, and inhibition of the activity or expression of this enzyme increased the surface expression levels of TβR1, as well as TGFβ-induced Smad3 and Akt activation [305]. ADAM17, via the shedding of the TβR1 ectodomain, is therefore, a negative regulator of TGFβ-signaling.

The intracellular cleavage of Notch induces the release of NICD (notch intracellular domain), which can then be translocated to the nucleus. Afterwards, NICD interact with a DNA-binding adaptor CBF1/RBPjk/Su(H)/Lag1, called CSL, to form a transcriptional activator complex [306]. This complex also recruits the adaptor protein Mastermind-like (MAML) and histone acetyltransferases HAT p300, favoring the chromatin opening and the activation of genes such as those encoding the hairy enhancer of split (HES) and HES-related with the YRPW motif (HEY). The half-life of NICD is controlled by its phosphorylation by cyclin-dependent kinase 8 (CDK8) and subsequent ubiquitination by E3 ubiquitin ligases, leading to its proteasome degradation [307,308].

Notch receptors, as well as their ligands, can be expressed in bone-forming cells and bone-resorbing cells [309,310,311,312]. For example, using flow cytometry analyses, Sekine et al. found that the Notch1 and Notch2 receptors are expressed in human osteoclast precursors (adherent cells isolated from human peripheral blood mononuclear cells), while Notch3 expression requires M-CSF (50 ng/mL) pre-treatment for 3 days. The expression of Notch1, Notch2, and Notch3 is maintained during the osteoclast differentiation process [311]. However, a low level of their ligand DLL1 protein is observed in osteoclast precursors, after stimulation by RANKL for 3 days, while JAG1 is constitutively expressed [311].

The role played by Notch in both osteoclastogenesis, as well as osteoblast differentiation, remains controversial due to discrepancy in the results obtained by several studies due to the experimental design, cell source, and operating conditions [311,313,314,315].

For example, Yamada et al. found that osteoclastogenesis, as shown by the TRAP positive cells, is decreased when precursors from the bone marrow, spleen, and peritoneal cavity are cultured on plates coated with human DLL1 for 6 days, with RANKL (25 ng/mL) and M-CSF (50 ng/mL). This inhibition depends on the tissue source of the osteoclast precursors varying from 23% to 100% for the bone marrow and the peritoneal cavity, respectively [313]. In contrast, Sekine et al. observed that blockade of DLL1 with specific antibodies inhibits osteoclastogenesis of both murine (bone marrow) and human (peripheral blood mononuclear cells) osteoclast precursors [311]. In fact, these apparent discrepancies can be due to the biphasic role of the Notch pathway in osteoclastogenesis and osteoclast maturation [310]. Indeed, Ashley et al. found that early activation of the Notch pathway in murine osteoclast precursors can suppress osteoclastogenesis, while Notch enhances the maturation and function of the committed osteoclast precursors [310]. Interestingly, inhibition of Notch in the murine myeloid lineage via a dominant negative MAML reduces the osteoclast function both in vitro and in vivo. However, it does not affect the osteoblast–osteoclast coordinated activity, which might help develop a promising therapeutic approach in fracture healing [316].

Several studies also highlighted the favoring role of the Notch pathway in osteoblast differentiation induced by BMPs [312,317], while others found a synergistic Notch/BMP effect on proliferation of multipotent progenitors [275]. For example, Cao et al. recently found that murine C2C12 myoblasts cultured in BMP-9 conditioned medium (collected 48 h after infection of HCT116 cells by Ad-BMP9) had less *Bglap* transcripts (Osteocalcin) in the presence of the Notch pathway inhibitors (Ad-dominant negative Notch1 and DAPT, γ-secretase inhibitor), as compared to BMP-9 alone [317]. The cell treatment by Ad-DLL1 for 36 h also enhances the level of phosphorylated Smad1/5/8 induced by BMP-9 conditioned medium in both C3H10T1/2 cells and C2C12 myoblasts. In fact, DLL1 might control BMP-9-induced osteoblastic differentiation through regulation of ALK2 expression [317]. In contrast, Wang et al. found that NICD overexpression inhibits the osteoblastic differentiation of C3H10T1/2 cells induced by AdBMP-9. NICD overexpression does not affect the levels of both total and phosphorylated Smad1/5/8, while it induces the suppression of JunB mRNA and protein [275].

## 4. Effect of TGF-β Superfamily on Bone Homeostasis and Disease

### 4.1. The Role Played by Members of TGF-β on Osteoblast and Osteoclast Differentiation

#### 4.1.1. Osteogenic Differentiation

The members of the TGF-β superfamily play a crucial role in the balance between bone formation and resorption. Indeed, the ability of the members of the TGF-β superfamily, especially BMPs such as BMP-2, BMP-4, BMP-6, BMP-7, and BMP-9, to induce the osteogenic differentiation of MSCs in vitro and bone formation in vivo is well documented [150,151,153,154]. However, the treatment of MSCs from various species by BMPs can be performed using AdBMPs, chemically modified ribonucleic acids, or human recombinant (rh) BMPs, rendering the comparison of the experimental data difficult [151,318,319].

Interestingly, several studies observed a higher osteogenic potential for BMP heterodimer compared to homodimer [320,321,322,323]. For example, rhBMP-2/BMP-7 heterodimer (rhBMP2/7) at a low-dose (5–50 ng/mL) drastically enhanced the differentiation of murine MC3T3-E1 preosteoblasts into mature osteoblasts, compared to rhBMP-2 or rhBMP-7 homodimer alone. The mineralization induced by rhBMP2/7 at 50 ng/mL is around 10- and 35-fold higher than that induced by rhBMP-2 and rhBMP-7, respectively, as shown by the alizarin red staining of the calcium deposition at 4 weeks [320]. Zhang et al. recently observed that rhBMP-2/7 at 50 ng/mL induces a higher deposition of calcium, as shown by the alizarin red staining, than rhBMP-2 and rhBMP-7 in MC3T3-E1 preosteoblasts, after incubation for 3 weeks. However, in this study, the BMP heterodimer and homodimers were added to an osteogenic differentiation medium containing 100 nM dexamethasone, 0.2 mM ascorbic acid, and 10 mM beta-glycerophosphate. rhBMP-2/7 also induced a similar mineralization than both homodimers in human adipose stem cells, suggesting a “cell-specific pattern” of BMP heterodimer efficiency [324]. In addition, collagen sponges with 3 µg rhBMP-2/7 implanted in dorsal muscles of rat, promote a higher bone formation than those with rhBMP-2 or rhBMP-7 (3 µg), as shown by the bone volume (microCT):T2 high volume (MRI) ratio [322].

#### 4.1.2. Osteoclastogenesis

The member of the TGF-β family can act on osteoclast progenitor proliferation, osteoclastogenesis, bone resorption activity, as well as survival of mature osteoclasts through direct or indirect (via osteoblast/osteocytes secreted factors) mechanisms (Table 2) [59,171,325].

It was shown that BMP-9 (50 ng/mL) alone can increase the proliferation of mouse spleen macrophages after 3 days [265]. However, BMPs can also promote RANKL-induced osteoclast progenitor proliferation. For example, in the presence of rhRANKL (50 ng/mL), both rhBMP-2 and rhBMP-7 (from 5 to 200 ng/mL) increase the proliferation of RAW264.7 cells after 3 days, compared to the cells treated with rhRANKL alone [326].

**Table 2:** Effect of the member of TGF-β superfamily on osteoclast differentiation and function.

Members of TGF-β Superfamily	Experimental Conditions	Impact on Gene and Protein Expression	Impact on Osteoclast Function	Refs
**TGF-β/Nodal/Activin family**				
TGF-β1	***Cells***: Murine RAW264.7; ***Treatment***: M-CSF (20 ng/mL), RANK-L (50 ng/mL) and TGF-β1 (0.1 to 20 ng/mL); ***Time***: 2-7 days	TGF-β1 dose dependently ↑ *TNFRSF11A* (RANK) at 48 h TGF-β1 5 ng/mL ↑ RANK protein amount after 3 days TGF-β1 dose dependently ↑ both *CTR* and *VTR* mRNA levels at day 7	TGF-β1 dose dependently ↑ number of TRAP+ multinucleated cells (plateau at 1 ng/mL)	[327]
	***Cells***: murine primary osteoblasts co-cultured with spleen cells; ***Treatment***: 1,25(OH)2D3 (10 nM) plus Dex (100 nM) with or without rhTGF-β1 (0.3 to 10 ng/mL), M-CSF ((25 ng/mL), RANKL (50–200 ng/mL); ***Time***: 7 days	N.A.	TGF-β1 dose-dependently ↓ osteoclast formation (TRAP+ cells) in the presence of 1,25(OH)2D3 plus dexamethasone. TGF-β1 dose-dependently ↑ RANKL-induced osteoclastogenesis (TRAP+ cells) of M-CSF stimulated spleen cells cultured alone. RANKL/TGF-β effect is inhibited by OPG (100 ng/mL).	[328]
	***Cells***: Marrow-derived osteoclasts precursors co-cultured with ST2 stromal cells; ***Treatment***: TGF-β1 (2 × 10^−5^ to 2 ng/mL)	N.A.	Biphasic effect of TGF-β1 on osteoclast differentiation: ↑ number of TRAP+ multinucleated cells at 1 × 10^−4^ ng/mL Complete inhibition at 2 ng/mL	[329]
	***Cells***: marrow and spleen cells (osteoclast precursors); ***Treatment***: ascorbic acid (7 × 10^−3^ M) and TGF-β1 (2 × 10^−5^ or 1 ng/mL), M-CSF (25 ng/mL) and RANKL (30 ng/mL).	N.A.	Only TGF-β1 at 1 ng/mL ↑ number of TRAP+ multinucleated cells (spleen cells). TGF-β1 dose dependently ↑ number of TRAP+ multinucleated cells (marrow cells)	
	***Cells***: human mononuclear leukocytes from umbilical cord blood differentiated in osteoclasts; ***Treatment***: rhTGF-1 (0.1–1 ng/mL)	TGF-β1 ↑ pERK1/2, phosphorylated p38 and pSmad 2 TGF-1 ↑ amount of pro-apoptotic proteins (Bax/Bim). TGF-β1 ↑ expression of Bim through Smad 2.	TGF-β1 dose-dependently ↑ apoptosis of human osteoclasts through caspase 9	[330]
	***Cells***: monocytes from normal human peripheral blood; ***Treatment***: 20 ng/mL M-CSF for 2 days and then RANKL (40 ng/mL) for an additional 6 days with or without TGF-β1 (10 ng/mL); ***Time***: 8 days	In the presence of M-CSF/RANKL: TGF-β1 ↑ Endoglin expression (mRNA and protein) compared to M-CSF/RANKL control. TGF-β1 ↓ levels of mRNA encoding NFAT-c1, TRAP and Cathepsin K. TGF-β1 ↓ levels of mRNA encoding RANK and MMP-9 through Smad1 activation	In the presence of M-CSF/RANKL: TGF-β1 ↓ number of TRAP+ multinucleated cells in a Smad1 dependent manner TGF-β1 inhibits osteoclastogenesis only when added within 48 h TGF-β1 ↑osteoclastogenesis through a Smad3 dependent manner	[325]
TGF-β2	***Cells***: marrow and spleen cells; ***Treatment***: ascorbic acid and TGF-β2 (2 × 10^−5^ to 2 ng/mL) with or without M-CSF (25 ng/mL), RANKL (30 ng/mL).	N.A.	TGF-β2 biphasic effect on osteoclast differentiation: A maximal number of TRAP+ multinucleated cells at 2 × 10-4 ng/mL No TRAP+ cells at 2 ng/mL	[329]
Activin A	***Cells***: murine bone marrow cells (BMC); ***Treatment***: rhM-CSF (20 ng/mL) and rhRANKL (40 ng/mL) with or without rh activinA (50 ng/mL); ***Time***: 4 days **Cells**: Murine monocyte/macrophage cell line RAW264.7; ***Treatment***: rhRANKL (40 ng/mL) with or without rh activinA (50 ng/mL); ***Time***: 2, 3, 4 or 7 days	ActivinA ↑ RANKL-induced NFATc1 expression in both BMC and RAW264.7 via Smad2 phosphorylation ActivinA ↑ RANKL-induced osteoclastogenic gene (TRAP, OC-STAMP and Cathepsin K) expression in RAW264.7 at 3 days	ActivinA ↑ differentiation of both BMC and RAW264.7 in osteoclasts (as shown by TRAP+ cells at 4 and 7 days, respectively) in the presence of M-CSF and RANKL	[184]
**BMP/GDF family**				
BMP-2	***Cells***: murine primary osteoclast; ***Treatment***: 10 ng/mL of M-CSF for 3 days before adding 30 ng/mL of RANKL with or without BMP-2 (30 ng/mL) for 5 days	BMP-2 ↑ RANKL-induced genes encoding osteoclast markers (NFATc1, TRAP, DC-STAMP, cathepsin K and ATP6v0d2) at day 3 BMP-2 plus RANKL had no effect on RANKL or OPG expression at day 3	BMP-2 from day 3 to day 4 ↑ RANKL-induced osteoclast formation as shown by an increase in TRAP+ multinuclear cells Suppression of BMPRII expression by specific shRNA inhibits osteoclastogenesis	[331]
BMP-2	***Cells***: bone marrow mononuclear cells incubated ***Treatment***: 20 ng/mL of M-CSF for 4 days, followed by another 5 days with 20 ng/mL M-CSF and 50 ng/mL of RANKL with or without BMP-2 or BMP-7 at 100 ng/mL.	BMP-2 ↑the amount of pSmad1/5/9 through ALK2 and ALK3 BMP-2 via Smad activation ↑ NFATc1 protein levels and its nuclear translocation in osteoclasts	BMP-2 alone had no effect on osteoclast differentiation BMP-2 ↑ RANKL-induced osteoclastogenesis as shown by TRAP+ cells (with three or more nuclei) at day 5 BMP-2 plus RANKL ↑ the area of demineralized pits on OsteoAssay surface plates	[59]
BMP-7		BMP-7 ↑ the amount of pSmad1/5/9 through ALK2 BMP-7 via Smad activation ↑ NFATc1 protein levels and its nuclear translocation in osteoclasts	BMP-7 alone had no effect on osteoclast differentiation BMP-7 ↑ RANKL-induced osteoclast differentiation at day 5 BMP-7 plus RANKL ↑ demineralization activity	
BMP-9	***Cells***: human mononuclear leukocytes from umbilical cord blood are differentiated in osteoclasts; ***Treatment***: Opti-MEM media supplemented with 2% FBS, 25 ng/mL M-CSF and 100 ng/mL of RANKL with or without BMP-9 (50 or 150 ng/mL)	BMP-9 acts via BMPR-II receptor to activate ERK1/2 pathways ↓ of BMPR-II by siRNA prevents bone resorption	In the presence of M-CSF/RANKL: No effect of BMP-9 on osteoclast formation (no change in % of multinucleated cells expressing RANK or CTR) BMP-9 ↑ bone resorption (30–40%) BMP-9 (50 ng/mL) protects osteoclasts from apoptosis by ↓ the % of cleaved caspase 9 and its activity	[171]
Myostatin	***Cells***: Bone marrow–derived macrophages ***Treatment***: 50 ng/mL M-CSF for 72h. Then cells are incubated for 4–6 days with M-CSF (50 ng/mL) and RANKL (50 ng/mL) with or without myostatin (30 ng/mL)	Myostatin ↑ RANKL-induced expression of NFATc1; integrin αv, integrin β3, DC-STAMP and CTR Myostatin activates Smad2 to enhance RANKL-induced osteoclastogenesis NFATC1 and pSmad2 can interact together favoring their nuclear translocation	No effect of Myostatin alone on osteoclast formation, apoptosis, and proliferation Myostatin + M-CSF/RANKL ↑ osteoclastogenesis (3.8-fold more osteoclasts after 4 days compared with M-CSF/RANKL control) ALK4/ALK5/ALK7 inhibitor ↓ number of osteoclasts	[332]

↓: Decrease; ↑: Increase; N.A.: Not available.

Furthermore, several studies showed that some members of the TGF-β superfamily promote RANKL-induced osteoclast differentiation (Table 2). For example, Itoh et al. found that RANKL is required to observe any osteoclast differentiation of mouse bone marrow macrophages in the presence of rhBMP-2 (300 ng/mL), since adding rhOPG prevents osteoclastogenesis [333]. In the same way, both rhBMP-2 and rhBMP-7 favor the osteoclastogenesis of the RAW264.7 cells in the presence of rhRANKL (50 ng/mL). However, while rhBMP-2 (5–150 ng/mL), in the presence of RANKL, dose dependently increases the calcium phosphate resorption area compared to rhRANKL alone, rhBMP-7 induces less bone resorption at 150 ng/mL than at 5 ng/mL, after 7 days [326].

The rhBMP-2/7 heterodimers (5–150 ng/mL) also enhance the RANKL-mediated osteoclastogenesis [326]. The same observation was done using rhBMP-9, the cytokine at doses varying from 50 to 150 ng/mL, enhanced the osteoclast differentiation of mouse spleen macrophages induced by rhRANKL (100 ng/mL) [265]. The authors suggested that BMP-9 inhibits the intracellular ERK1/2 pathways to favor the osteoclastogenesis [265]. Using human mononuclear macrophages from umbilical cord, our research team also found that rhBMP-9 (50 and 150 ng/mL) in the presence of both rhM-CSF (25 ng/mL) and rhRANKL (100 ng/mL), significantly increases the bone resorption (around 40%) compared to the control. Nevertheless, this effect is not due to an increase in mature osteoclast formation, but an inhibition of their apoptosis [171]. Indeed, BMPs can also act on mature osteoclast function. Using mature osteoclasts purified (>99%) from rabbit bones, Kaneko et al. also found that among rhTGF-β, rhBMP-2, and rhBMP-4 (1, 10, 100 ng/mL), only rhBMP-2 and rhBMP-4 directly and dose dependently enhance the osteoclastic bone resorption after incubation for 18 h. This effect is not due to a change in the number of osteoclasts on dentine slices. In addition, rhBMP-2 at 100 ng/mL increases the mRNA levels of both cathepsin K and carbonic anhydrase II [172].

Some members of the TGF-β superfamily can also inhibit osteoclastogenesis [329]. Using marrow-derived osteoclasts precursors and ST2 stromal cells co-cultures, Karst et al. found that both TGF-β1 and TGF-β2 have a biphasic effect on osteoclast differentiation, increasing it at low concentration (1 × 10^−4^ or 2 × 10^−4^ ng/mL), while completely preventing it at high concentration (2 ng/mL) [329].

**Figure 4:**
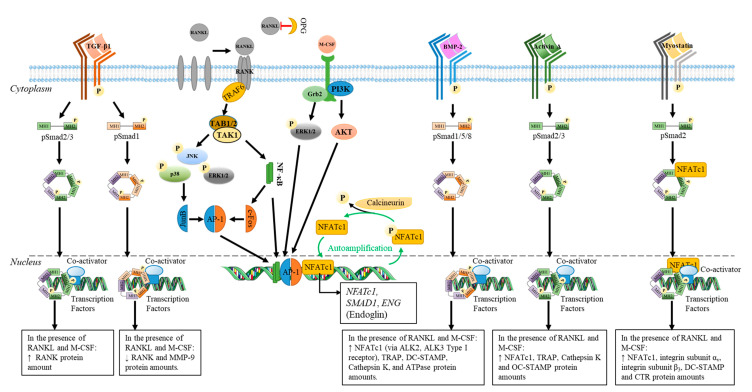
Crosstalk between TGF-β superfamily signaling and M-CSF/RANKL pathways to regulate osteoclast differentiation and function [59,184,325]. CTR: Calcitonin receptor; DC-STAMP: Dendritic cell–specific transmembrane protein; MMP: Matrix metalloproteinase; OC-STAMP: Osteoclast Stimulatory Transmembrane Protein; and TRAP: Tartrate-resistant acid phosphatase. The figure was created using Servier Medical Art. https://smart.servier.com.

Lee et al. also recently found that TGF-β1 (0.1, 1, 10 ng/mL) dose-dependently decreases the number of TRAP-positive multinucleated cells incubated for 2 days with 20 ng/mL M-CSF and then 40 ng/mL RANKL for 6 days [325]. However, TGF-β1 inhibits osteoclastogenesis only when added at the early stages of differentiation, within 48 h. It also decreases the mRNA levels of NFAT-C1, TRAP and Cathepsin K. Interestingly, TGF-β1 through Smad3 stimulation, favors RANK expression in human osteoclast precursors, while TGF-β1 through Smad1, suppresses it [325] (Figure 4).

### 4.2. Temporal Expression of the Members of TGF-β Superfamily during the Bone Fracture Healing Process

The members of the TGF-β superfamily were detected in all phases of the bone fracture healing, but their temporal expression patterns and amounts might vary in function of the animal model used [334,335,336]. Their effects are also coordinated with those of inflammatory cytokines, other growth factors (PDGF, VEGF, FGF1, and FGF2) and extracellular matrix proteins like type I collagen [337,338,339].

Cho et al. followed the mRNA levels of TGF-βs and BMPs in fractured mouse tibias for 4 weeks [334]. They found that TGF-β1 is already highly expressed before the fracture occurs. However, its transcript level slightly increases during the hematoma-inflammation formation phase, 1 day after the fracture. It then remains highly expressed within 28 days, but at a level similar to that detected before the fracture. In contrast, *TGFB2* and *TGFB3* genes are only expressed after the fracture and in a smaller amount than TGF-β1 mRNA. Both TGF-β2 and TGF-β3 transcript levels remain high between days 1–7 and days 1–21, respectively. Thus, while TGF-β2 is mainly expressed during the fibrocartilaginous callus formation, TGF-β3 appears to act during both fibrocartilaginous and bony callus formation (bone repair phase). Indeed, the gene (*COL2A1*) encoding type II collagen specific to the cartilaginous matrix is highly expressed at day 7, while the expression of gene (*COL1A1*) encoding type I collagen specific to the osteoid matrix reaches a maximum, at days 14 and 21 [334].

It was also confirmed that human fracture hematoma contains a high concentration of TGF-β1 [340]. Interestingly, Zimmermann et al. suggested that the amount of TGF-β1 in human serum can be used as an indicator of non-union fracture. They found that a drastic decrease in TGF-β1 serum level occurs at 4 weeks, in patients suffering from delayed fracture healing, compared to patients with normal healing [341]. However, Sarahrudi et al. did not find any difference in the concentration of TGF-β1, in serum from patients with normal and impaired fracture healing, suggesting that further comparative studies must be performed to confirm TGF-β1 as a potential marker of non-union fractures [340]. In addition, Burska et al. recently suggested that TGF-β2 and placenta growth factor might also be promising markers of human fracture healing [105].

Cho et al. also observed that among BMPs, BMP-2 is the earliest activated gene in fractured mouse tibias. The kinetic profiles of the BMP-2 transcripts reveal two peaks of similar intensity at day 1 (hematoma-inflammation formation phase) and day 21 (bone repair phase). The expression of genes encoding BMP-4, BMP-7, and BMP-8 reaches a peak between 14 and 21 days, corresponding to the bone repair phase [334]. In contrast, Cottrell et al. found that BMP-2 transcript level is almost 4-fold higher at 21 days than that at day 2 in the femur fracture calluses of female Sprague–Dawley rats. They also showed that only the expression of genes encoding for BMP-2 and BMP-4 changes over time. The highest mRNA level of BMP-2, BMP-4 (as well as TNF-α), at 21 days, is in accordance with an increase in osteoclast resorption activity during callus bone remodeling [336]. The expression of Ser/Thr kinase receptors can also vary during bone fracture healing [342]. Furthermore, bone cells involved in the fracture healing process can differently express the member of the TGF-β superfamily. Using human fracture callus specimen (5 patients between 15 and 44 years old), Kloen et al. found that the staining of BMP-2 and BMP-4 is less intense in the osteoblasts than that of BMP-7 [343]. In contrast, mature chondrocytes highly express BMP-2 and BMP-4. Both BMPs are also found in newly synthesized osteoid matrix. In addition, BMP-4 is not detected in osteoclasts, while some of them express BMP-7, BMP-2, and BMP-3. BMPs are also co-localized with their Ser/Thr kinase receptors and pSmad1 [343].

Age can affect the fracture healing and BMP gene expression profile obtained during the fracture healing process. Using a closed mid-diaphyseal fracture in 6 weeks and 1-year-old rats, Meyer et al. found that only younger rats present a fracture healing at 6 weeks [335]. The fracture induces the expression of BMP-2 in both younger and older rats. Nevertheless, BMP-2 transcripts level reaches a peak at 1 week in younger rats, while it is observed only after 2 weeks in older ones. In addition, BMP-2 mRNA level in the younger rats is higher than that observed in the older rats. BMP-4 and BMP-6 mRNA are detected before the fracture in younger rats. Again, both BMP-4 and BMP-6 transcripts reach a peak at 1 and 2 weeks for younger and older rats, respectively. These different mRNA kinetic profiles or transcript amount might explain the delayed fracture healing in older rats [335].

### 4.3. TGF-β Family Members and Bone Diseases

Abnormal TGF-β signaling and polymorphisms in TGF-β1 are involved in widespread human disorders, such as fibrosis and cardiovascular diseases, as well as hereditary or sporadic cancers. Various heritable developmental disorders in humans are caused by mutations in the TGF-β system [15,344]. Given the importance of TGF-β signaling in bone remodeling, particularly as a coupling factor between resorption and formation, it is not surprising that members of the TGF-β family are also implicated in metabolic bone diseases (osteoporosis) or bone malignancies (metastases, multiple myeloma). Similarly, a bone phenotype might be observed upon mutations of one of the genes encoding a member of the TGF-β family or of its receptors.

#### 4.3.1. TGF-β Signaling and Osteoporosis

Osteoporosis is a systemic bone disorder characterized by low bone mass and microarchitectural deterioration, with consequent bone fragility and an increase risk of fractures. Bone loss occurs in postmenopausal women as a result of an increase in the rate of bone remodeling, and an imbalance between bone resorption, which is higher than bone formation [345]. The large increase in bone resorption is related to an increase in osteoclastogenesis. Estrogens increase TGF-β secretion by osteoblasts, and this factor could be responsible for the estrogen-induced osteoclast apoptosis [346]. TGF-β reduce RANKL expression, and both TGF-β and estrogens increase OPG expression [347,348]. However, the effects of TGF-β1 are complex. If TGF-β1 decreases the RANKL:OPG ratio and inhibits the recruitment of osteoclasts, its effect on the mature osteoclast would be rather stimulating [349]. Furthermore, it was recently shown that adding RANKL to M-CSF-stimulated bone marrow mononuclear cells can increase the expression levels of genes encoding BMP-2 and BMP-7 within 1 day. The resulting secreted BMPs activate Smad1/5/9 promoting osteoclast fusion [59].

Genetic polymorphisms of members of the TGF-β family are associated with osteoporosis and low bone mass, such as polymorphisms in the genes encoding TGF-β, BMP2, and BMP4 [350].

#### 4.3.2. TGF-β Signaling and Osteogenesis Imperfecta

Osteogenesis imperfecta (OI) is an autosomal dominant form of osteoporosis most often caused by mutations in type I collagen genes (*COL1A1*, *COL1A2*). The altered quality of the bone matrix, composed mainly of type I collagen, could stimulate TGF-β signaling. In fact, the TGF-β produced by osteoblasts is secreted and included in the bone matrix in an essentially latent form. During osteoclastic bone resorption, this coupling factor is released and activated. However, the matrix environment of OI results in excessive TGF-β activation and signaling, which contributes to low bone mass [351]. TGF-β appears as a pathogenic factor and has become a therapeutic target in OI, with favorable effects of its blockade by neutralizing anti-TGF-β antibodies in two mouse models of OI, Crtap−/− and +/G610C mice, with increased bone mass [351]. However, in another OI model, Col1a1 Jrt/+ mice, which differ from the previous ones by a clear propensity to fractures, the administration of the same anti-TGF-β1 D11 antibody had no impact on bone mass, nor on the quality of the bone matrix [352].

#### 4.3.3. TGF-β Signaling in Bone Malignancies

- Bone metastases

In breast carcinoma metastases, osteolytic bone disease is observed in the vicinity of the tumor cells, where a vicious circle is created. Indeed, during osteolysis, growth factors such as TGF-β are released and these contribute to the growth of bone metastases, and TGFβ strongly stimulates the production of PTHrP by tumor cells [353,354].

- Multiple Myeloma

Multiple myeloma (MM) is a B cell malignancy characterized by the presence of an expanded monoclonal population of plasma cells secreting a monoclonal immunoglobulin in the bone marrow, and the development of an osteolytic bone disease [355].

Many osteoclast activation factors were identified in myeloma bone disease [356], among them TGF-β is present in the bone matrix and is released upon resorption. TGF-β stimulate the production of IL-6 and RANKL and the development of Th17 cells, thereby increasing osteolysis and decreasing bone formation. In preclinical models, blockade of TGF-β signaling by a type I receptor inhibitor [357], or by administration of a small peptide with a sequence derived from the latent form of TGF-β, which blocked TSP1–TGF-β binding (and thus TGF-β activation), reduced tumor burden, decreased bone resorption, and stimulated bone formation [358].

- Targeting Activin A in Myeloma

Activin A, produced after interaction of bone marrow cells with myeloma cells, stimulates osteoclastic resorption, and inhibits osteoblast formation. High levels of activin A, correlating with the extent of osteolysis and with poor survival, were reported in subjects with advanced MM [359]. In a mouse model of MM, the administration of an activin antagonist—a soluble form of the extracellular domain of the type IIA receptor of activin coupled to the Fc fragment of Ig (RAP-011)—decreased the number of osteolytic lesions, increased bone mass, and decreased tumor burden [360].

ACE-011 is a fusion protein composed of the extracellular domain of the human activin receptor type IIA linked to the Fc fragment of human IgG1, capable of binding activin. Administration of ACE-011 results in an increase in bone formation markers, and a decrease in bone resorption markers (phase I study in postmenopausal women) [361]. In a phase II study in multiple myeloma, the activin A antagonist (sotatercept or ACE-011), in combination with chemotherapy, was found to significantly increase bone mass [362].

- TGF-β Family in Monogenic Developmental Bone Diseases

Mutations in genes of BMP receptors are implicated in human skeletal disorders, such as *BMPR1B* encoding the BMPR-IB receptor in acromesomelic chondrodysplasia [363] and *ACVR1* encoding ALK2 in progressive fibrodysplasia ossifying (FOP) [364].

FOP, a rare genetic disorder with an incidence of one in two million, is characterized by progressive ectopic bone formation in soft tissue (heterotopic ossification (HO)) such as skeletal muscle, tendon, ligament), either spontaneously or after trauma [365] (for review see [366]). *ACVR1* mutations do not affect the expression of ALK2 but result in an increase in ALK2 gain of function related to a R206H substitution in the intracellular GS-rich domain of the receptor linked to 95% of the patients [364,367]. Thus, the BMP signal transduction in FOP cells, through the canonical (Smad) and non-canonical (MAPK) pathways, is overactive, leading to the transcription of targeted genes [368,369]. Using an in vivo model of injury-induced HO (Acvr1R206H/+ knock-in mouse), Haupt et al. found that injured tissue at early stages of repair, is stiffer, favoring permissive condition to HO formation. The small Rho GTPase mechano-signaling pathway (Rho/ROCK) is also over-activated in the *Acvr1* R206H/+ cells and might act synergistically with BMPs, to favor osteogenesis [370]. It was also shown that the R206H substitution rendered ACVR1 responsive to activin A, which generally antagonize BMP signaling through ACVR1 but cannot normally induce bone formation. Inhibition of activin A in a knock-in model of ACVR1 R206H, using a blocking antibody, completely inhibits the development of HO [371].

Camurati–Engelmann disease is a progressive diaphyseal dysplasia, presenting with a characteristic thickening of the long bone diaphysis, mainly femurs, with an increase in bone density. Camurati disease is autosomal dominant, and mutations in *TGFB1* encoding TGF-β1 were reported, mostly located in the latency-associated domain of TGF-β1, and suggest an increase in TGF-β signaling [372].

Finally, somatic mutations in *SMAD3* were described in Melorheostosis, a sporadic bone disease. Melorheostosis is a sclerosing bone dysplasia, characterized by cortical hyperostosis, affecting endosteal and periosteal surfaces, with a typically asymmetric distribution, and a classic “dripping candle wax” radiological appearance. *SMAD3* mutations increase TGF-β signaling and stimulate osteogenesis [373]. Mutations in *MAP2K1* was already reported in this disease by the same authors, with a different clinical and histological profile [374].

## 5. The Use of Members of the TGF-β Superfamily in Clinical Application and Their Potential Adverse Effect

The use of BMPs for therapeutic purposes necessarily involves large-scale production to meet market needs. The extraction and purification of small quantities of BMPs began from demineralized cadaveric bovine bone sources, a technique that required a very long production time and a contribution of several kilograms of bone at a very high cost (several kg of bone = µg of purified BMPs) [375]. Subsequently, this procedure was replaced by the molecular cloning of coding sequences (cDNA) for members of the BMPs family expressed in different recombinant systems (Bacteria: *Escherichia coli*; Yeast based: Pichia pastoris; Baculovirus/insect cell system (Baculovirus Expression Vector Systems: BEVS); and Mammalian cells: Chinese hamster ovary (CHO)) [137,376,377,378]. This strategy made it possible to obtain a higher yield of proteins and a better reproducibility, reliability, and safety of the BMPs produced. However, in addition to large-scale production, BMPs must be expressed in a system that ensures biological activity without immunogenicity, so that they can be used for therapeutic purposes. It is necessary to use eukaryotic expression systems that are capable of inducing glycosylation of BMPs [379]. Indeed, this glycosylation is of critical importance, since it deeply affects the biological activity, the elimination, and recognition by the body (antigenicity) of proteins [380]. Thus, genetic engineering that allows the cloning of cDNA in CHO cells is used for the production of clinical quality BMPs in large quantities [381].

To date, the FDA approved the use of two types of rhBMPs (rhBMP-2 and rhBMP-7) associated with commercial delivery systems as an alternative to autologous bone graft in specific orthopedic applications (InFUSE^®^ and OP-1^®^) [382]. RhBMP-2 was approved for use in human spine surgery on a collagen sponge support absorbable by the InFUSE^®^ system (Medtronic Sofamor Danek, Inc.) [383,384,385]. However, rhBMP-7 only received a “Humanitarian Device Exemption” in 2004, for use in compromised patients that require revision of posterolateral (intertransversed) lumbar arthrodesis, for whom bone removal is not possible [386,387]. The rhBMP-7 was used in combination with bovine bone collagen (OP-1 Implant^®^) (Stryker Biotech/Olympus) and with carboxymethylcellulose (OP-1 Putty^®^) (Stryker Biotech/Olympus).

Recent studies demonstrated the benefits of using rhBMP-2 and rhBMP-7 for orthopedic treatments and surgeries, particularly in spinal fusion, lumbar fusion, and tibial fracture repair [388,389,390,391] (Table 3). While BMP-7 treatment results in decreased operating time for lumbar fusion, rhBMP-2 decreases the risk of re-operation and improves the success of lumbar fusion and bone union of tibial fractures [389,390,391]. However, many studies also reported complications related to the use of BMP-2, especially for cervical fusions associated with pain, wound infections, dysphagia, and hoarseness, leading to a large increase in hospital costs [392,393,394]. In 2008, the FDA warned the public health concerning the use of BMP-2 in anterior cervical fusion applications. Furthermore, OP-1 devices that were sold initially by Stryker and then by Olympus in 2010, are not produced anymore, despite the fact that BMP-7 is still used in several clinical trials (Table 3). Indeed, the commercial scaffolds used to deliver rhBMPs to the surgical site are mainly composed of collagens. These proteins can be rapidly degraded in the body by proteolysis during the first days, after the operation, due to the inflammatory response induced by the surgery [381,382]. To counterbalance the intense proteolytic activity at the implantation site, the doses of rhBMP used are also often very high (AMPLIFY ™, rhBMP-2, 40 mg), particularly in commercial applications for spinal fusions, and are associated with a higher risk of cancer and adverse effects [392]. The use of increased doses of BMP-2 for spinal repair surgeries is associated with overactivation of osteoclasts, leading to complications such as osteolysis and graft subsidence [395,396,397]. The addition of bisphosphonate in combination with BMP-2 treatment, can reduce bone resorption, while promoting new bone formation [398].

Furthermore, higher osteoinductive activity of BMP-6 and BMP-9, compared to BMP-2 or BMP-7, make them promising candidates for promoting bone repair or filling, as shown by several recent studies [399,400,401]. For example, a recent randomized, double-blinded, placebo-controlled phase I/II clinical trial revealed that autologous blood coagulum combined with rhBMP-6 (1.0 mg/10 mL) promoted bone healing in patients with high tibial osteotomy [401].

Therefore, the heterogeneity of the experimental approach (type of BMP used, doses, and mode of administration) and the diversity of existing bone repair applications made the conclusions of most studies unclear [402]. Future clinical trials should be randomized, double-blind, and properly designed, in order to present a better understanding of the real potential of BMP applications [391,402]. A big challenge in the clinical application of rhBMP is to improve the properties of the delivery systems, in order to have a better control over the spatial and release cytokine kinetics in vivo.

**Table 3:** The use of rhBMP-2/rhBMP-7 in bone clinical application and their potential adverse effect [381].

rhBMP	Clinical Application	Methodology	Dose	Conclusion and Adverse Effect	Refs
BMP-2	Anterolateral interbody fusion	3105 patients (anterolateral interbody fusion: 2000–2012) from 14 trials (PubMed database and FDA approval document)	2.1–18 mg	Safe under FDA-approved recommendations (i.e., one-level anterolateral interbody fusion surgery with an LT-cage); Low complications (subsidence, cancer, infection); Equal efficiency (fusion rate, pain disability, patient satisfaction, risk of re-operations) between BMP-2, allogenic or autologous bone graft; Safety and effectiveness of BMP-2 in off-label use: not established.	[388]
BMP-2	Spinal fusion surgery/degenerative disc disease (control: iliac crest bone graft (ICBG))	1408 patients (spinal fusion: 1997–2012) from 12 trials (mostly sponsored by Medtronic)	Infuse^®^ (1.5 mg/mL) Amplify^®^ (2.0 mg/mL)	↑ early postsurgical pain compared with ICBG; Evidence of ↑ cancer incidence is inconclusive; ↑ fusion rates at 24 months.	[403]
BMP-2	Spinal fusion (control: bone graft)	1984 patients (spinal fusion: 1996–2012) from 13 trials (sponsored by Medtronic and Norton Healthcare)	0.6 to 16.8 mg (11 trials); 15.0 to 63.0 mg (5 trials of posterolateral lumbar fusion studies)	↑ complication in anterior cervical fusion: wound complication and dysphagia.; No proven clinical advantage over bone graft in spinal. fusion: May be associated with important harms (retrograde ejaculation and urogenital problems); ↑ cancer risk at 24 months.	[394]
BMP-2	Spinal fusion	55,862 patients (spinal fusion: 2004–2007) from the Scoliosis Research Society database (BMP used in 21% of all spinal fusions)	N.A.	↑↑ incidence of complications and wound infections in anterior cervical fusions; Not associated with ↑ complications in thoracolumbar and posterior cervical fusions.	[393]
BMP-2	Spinal fusion	780 patients (1995–2010) from 13 trials (sponsored by industry).	0.6–40 mg	↑↑ complications and adverse events in spinal fusion; Possible study design bias in the original trials: risk of adverse events around 10 to 50 fold that of the original estimates reported in publications sponsored by industry; Higher doses of BMP-2: associated ↑ risk of new malignancy.	[395]
BMP-2	Lumbar and lumbosacral fusion	129 patients (2000–2008) from the New York Harbor Health Care System Manhattan Veterans Administration operating room record	12 and 24 mg	Higher doses of rhBMP2 in lumbar and lumbosacral fusion: may ↑ risk of renal insufficiency.	[404]
BMP-7	Single-level lumbar fusion (control: ICBG)	539 patients (2002–2016) from 5 trials (PubMed, EMBASE, Scopus, and the Cochrane Collaboration Library databases)	3.5 mg of (rh)BMP-7 (Osigraft or Putty) per side	Shorter operation times; No additional beneficial effect (clinical success, revision rates and duration of hospitalization) between BMP-7 and ICBG; ↓ lumbar fusion rate (in instrumented posterolateral fusion).	[389]
BMP-2 and/or BMP-7	Lumbar fusion	2185 patients (2000–2016) from 21 trials	12–48 mg	↑ lumbar fusion success rate (BMP-2) and ↓ risk of re-operation; No difference in complication rate between BMPs and ICBG.	[390]
BMP-2 and/or BMP-7	Treatment of fractures, non-union and osteonecrosis	3324 patients (1601 fracture, 1654 non-unions and 69 osteonecrosis: from 2000 to 2016) from 43 trials (PubMed database)	Inductos^®^ (0.75, 1.5 or 2.0 mg/mL); Infuse^®^(1.5 mg/mL); OP-1 Stryker (3.3 and 3.5 mg/mL); Osigraft (3.5 mg/mL)	Controversial clinical evidence (fractures, non-union, and osteonecrosis); Preliminary knowledge and few low quality reports; Positive findings in many studies, but mixed efficacy and adverse events in overall literature; Unclear conclusions (heterogeneity of studies: different BMPs, doses and delivery method for each bone pathology).	[402]
BMP-2 and/or BMP-7	Tibial fracture and nonunion	1113 patients (tibial fracture and nonunion: 1997 to 2011) from 8 trials (MEDLINE, EMABSE, BIOSIS and Cochrane central data bases)	3.5, 6 or 12 mg	↑ effectiveness of bone union and ↓ risk of re-operation (tibial fractures); Equal efficiency (bone union, infection, or re-operations rate) between BMPs and autologous bone graft to treat tibial fractures non-union.	[391]
BMP-2 and BMP-7	Spinal fusion	941 patients from 7 trials from Pubmed, Cochrane, National Guideline Clearinghouse databases, FDA safety summaries (2012)	4–40 mg	↑ cancer risk dependent on the dose of BMP used.	[405]

↓: Decrease; ↑: Increase; ↑↑ Strongly increase; N.A.: not available.

## 6. Conclusions

The members of the TGF-β superfamily are potent regulators of homeostasis and bone repair that act on cell proliferation, osteogenic differentiation, osteoclastogenesis, and osteoblast/osteoclast balance. A good understanding of their characteristics, biological functions, canonical, and non-canonical signaling pathways, as well as their respective regulation is essential for allowing their safe use in the restoration of injured or diseased skeletal tissues. Despite their incredible potential for improving bone repair, they are linked to various postoperative complications (wound infections, dysphagia, tissue damage, cancer risks, etc.) pushing the FDA to limit their use to specific orthopedic application. The reduction of the applied BMP doses, as well as the development of delivery systems allowing a good control of their spatial and temporal release, seem to be the keys to their safe and effective clinical use. However, future well-designed studies are needed to get a better picture of the advantages and drawbacks associated with the use of the TGF-β superfamily members in clinical applications.

## Figures and Tables

**Table 1:** Type I and type II receptors, TGF-β, and signaling in multipotent stem cells, osteoblasts, and osteoclasts [162,163,173,174,175].

Type I Receptor	Type II Receptor	TGF-β ligands	Signaling Pathway Activation in Osteoclast Precursors and Mature Osteoclasts	Signaling Pathway Activation in Stem Cells and Osteoblast
**TGF-β/Nodal/Activin family**				
TβRI (ALK5); ALK1	TβRII	TGF-β1	↑ pSmad2/3 (human M2 monocyte-derived macrophages; 10 ng/mL) [176]; ↑ Smad1/5 (human M2 monocyte-derived macrophages; concentration is not specified) [174]; ↑ Wnt10b and crosstalk between Smad2/3 and canonical Wnt signaling (murine osteoclasts; 2 ng/mL) [177]	↑ pSmad2/3 (L6E9 myoblasts; < 0.01 ng/mL) [178]; ↑ pSmad1/5 (L6E9 myoblasts; <1 ng/mL) [178]; Crosstalk with Akt (early phase of osteoblast differentiation MC3T3-E1; 0.1 ng/mL) [179]; MAPK: ↑ pERK1/2, ↑pp38, ↑pJNK (MC3T3-E1; 2.5 ng/mL) [180]; Canonical Wnt: ↑ β-catenin via ALK5, Smad3 receptor and PI3K (hMSC; 1 ng/mL) [181]
		TGF-β3	N.A.	↑ pSmad2/3 (mouse embryonic palatal mesenchymal cells; 10 ng/mL) [182]; MAPK: ↑ pERK1/2 (human mesenchymal stem cells; 10 ng/mL) [183]
ActRIb (ALK4)	ActRIIA; ActRIIB	Activin A	↑ c-fos (murine macrophages RAW264.7; 50 ng/mL of activin A with 40 ng/mL of RANKL) [184]; ↑ pSmad2/3 (RAW264.7; 50 ng/mL) [184]; ↑ pSmad2/3 (murine bone marrow macrophages; 100 ng/mL) [185]; MAPK: ↑ pp38 and ↑ pERK1/2 (murine bone marrow macrophages; 100 ng/mL of activin A with 50 ng/mL of M-CSF) [185].	↑ pSmad2/3 (human endometrial stromal cells; <20 ng/mL) [186]
**BMP/GDF family**				
BMPRIA (ALK3); BMPRIB (ALK6); ActRI	BMPRII; ActRIIA; ActRIIB	BMP-2	↑ Smad1/5/9 (murine bone marrow mononuclear cells; 100 ng/mL) [59]; MAPK: ↑ pERK and ↑ pp38 (minimal) (murine bone marrow mononuclear cells; 100 ng/mL) [59]; ↑ pAkt (murine bone marrow mononuclear cells; 100 ng/mL) [59]; ↑ pSmad1/5 (osteoclasts precursor fusion; 30 ng/mL) [187]; MAPK: ↑ pp38 (osteoclasts precursor fusion; 30 ng/mL) [187]	↑ pSmad1/5 (C2C12 cells; 100 ng/mL) [188]; ↑ pSmad1/5 (human stem cells from the apical papilla; 100 ng/mL) [189]; ↑ pERK1/2 (human stem cells from the apical papilla; 100 ng/mL) [189]; ↑ pSmad1/5 (MC3T3-E1 preosteoblasts; 0.38 nM) [190,191]; MAPK: ↑ pERK1/2, ↑ pp38 (MC3T3-E1 preosteoblasts; 0.38 nM) [190,191]; ↑ pAkt (MC3T3-E1 cells; < 0.1 nM) [192].
		BMP-4	N.A.	↑ pSmad1/5 (C2C12 cells; 200 ng/mL) [193]
BMPRIA (ALK3); BMPRIB (ALK6); ALK2; ALK1	BMPRII; ActRIIA; ActRIIB	BMP-5	N.A.	↑ pSmad1/5 (human embryonic stem cells; 100 ng/mL) [194]; MAPK: ↑ pp38 (human embryonic stem cells; 100 ng/mL) [194]; MAPK: ↓ pp38 (murine osteoblasts—differentiated MC3T3-E1 cells; BMP-5 siRNA 40 nmol/mL) [195]
		BMP-6	↑ pSmad1/5 (rat and human granulosa cells; 100 ng/mL) [196].	↑ pSmad1/5 (C2C12 cells; 200 ng/mL) [193]; ↑ pSmad1/5 (human embryonic stem cells; 100 ng/mL) [194]; ↑ pSmad1/5 (MC3T3-E1 cells; 300 ng/mL) [175].
		BMP-7	↑ pSmad1/5/9 (murine bone marrow mononuclear cells; 100 ng/mL) [59]; MAPK: ↑ pp38 (murine bone marrow mononuclear cells; 100 ng/mL) [59]; ↑ pSmad1/5 (rat and human granulosa cells; 100 ng/mL) [196].	↑ pSmad1/5 (C2C12 cells; 1000 ng/mL) [193]; ↑ pSmad1/5 (human embryonic stem cells; 100 ng/mL) [194]; MAPK: ↑ pp38 (human embryonic stem cells; 100 ng/mL) [194].
ALK1 ALK2	BMPRII; ActRIIA; ActRIIB	BMP-9 (GDF-2)	↑ pSmad1/5 (human cord blood monocyte as osteoclast precursor; 150 ng/mL) [171]; MAPK: ↑ pERK1/2 (human cord blood monocyte as osteoclast precursor; 150 ng/mL) [171]	↑ pSmad1/5 (MC3T3-E1 cells; 0.38 nM) [190,191]; MAPK: ↓ pERK1/2, ↑ pp38, ↑ pJNK (MC3T3-E1 cells; 0.38 nM) [190,191]; ↑ pAkt (MC3T3-E1 cells; < 0.1 nM) [192]; ↑ pSmad1/5 (murine multipotent stem C3H10T1/2 cells; 10 ng/mL) [197]; MAPK: ↑ pp38 (C3H10T1/2 cells; 10 ng/mL) [197];
		BMP-10	N.A.	↑ pSmad1/5 (human embryonic stem cells; 100 ng/mL) [194]; MAPK: ↑ pp38 (human embryonic stem cells; 100 ng/mL) [194].
BMPRIA (ALK3); BMPRIB (ALK6)	BMPRII	BMP-15	↑ pSmad1/5 (immortalized human granulosa cells and human granulosa cell tumor cells; 100 ng/mL) [198]; ↑ pSmad1/5 (rat and human granulosa cells; 100 ng/mL) [196].	N.A.
BMPRIA (ALK3); BMPRIB (ALK6)	BMPRII; ActRIIA; ActRIIB	GDF-5/-6/-7	N.A.	MAPK: ↑ pp38 and ↑ pERK1/2 (chondrogenic mouse carcinoma cell line ATDC5; <10 ng/mL and 1000 ng/mL, respectively) [199]; ↑pSmad1/5 (C3H10T1/2 cells; Ad-GDF6) [200]; MAPK: ↑ pp38 (C3H10T1/2 cells; Ad-GDF6) [200].
ActRIb (ALK4)	ActRIIA; ActRIIB	GDF-8(myostatin)/-11	↑ pSmad2/3 (human hepatocellular carcinoma; Ad-GDF11) [201]	N.A.
ActRIb (ALK4)	ActRIIA; ActRIIB	GDF-10/BMP-3	N.A.	↑ pSmad 2/3 (murine C2C12 cells; 100 ng/mL) [202].

↓ Decrease; ↑ Increase; N.A.: Not available.

## Abbreviations

1,25-(OH)2D3	1α,25-dihydroxyvitamin D_3_
ActRIIA	Type II activin receptor
ActRIIB	Type IIB activin receptor
ALK	Activin receptor-like kinases receptor
ALP	Alkaline phosphatase
AMH/MIS	Anti-Müllerian hormone/Müllerian inhibiting substance
AMHRII	Anti-Mullerian hormone receptor type II
AP-1	Activator protein 1
APC	Adenomatous polyposis coli
BAMBI	Bone morphogenetic protein and activin membrane-bound inhibitor
BMMs	Bone marrow–derived macrophage
BMP	Bone morphogenetic proteins
BMPRII	Type II BMP receptor
BMU	Basic multicellular unit
BSP	Bone sialoprotein
CA2	Carbonic anhydrase enzymes
CAMKII	Calmodulin-dependent kinase II
CDK8	Cyclin-dependent kinase 8
CEBPα	CCAAT-enhancer binding protein α
CHO	Chinese hamster ovary
CKI	Casein kinase I
CRD	Extracellular N-terminal cysteine-rich domain
CREB	Cyclic AMP response element-binding protein
CSF-1	Colony stimulating factor 1
CTR	Calcitonin receptor
DAG	Diacylglycerol
DAP12	DNAX associated protein 12kD size
Dkk	Dickkopf
DLL	Delta-like
dpp	Drosophila decapentaplegic
DVL	Disheveled
ENG	Endoglin
ERK	Extracellular signal-regulated kinase
FGF	Fibroblast growth factor
FKBP12	FK506 binding protein of 12 kDa
FOP	Fibrodysplasia ossifying
FSH	Follicle-stimulating hormone
Fz	Frizzled
GDF	Growth differentiation factors
Grb2	Growth factor receptor bound protein 2
GSK3 β	Glycogen synthase kinase-3 β
GS motif	Gly/Ser rich motif
HAT	Histone acetyltransferases
HES	Hairy enhancer of split
HEY	HES-related with YRPW motif
HO	Heterotopic ossification
ID1	DNA binding protein 1
IGF	Insulin like growth factor
IL	Interleukins
IP3	Inositol-1,4,5-trisphosphate
I-Smad	Inhibitory Smad
JAG	Jagged
JNK	c-Jun amino (N)-terminal kinases
LAP	Latency associated peptide
LEF	Lymphoid enhancer-binding factor
LGR4	Leucine rich repeat containing G-coupled receptor 4
LRP5/6	Low-density-lipoprotein-related protein 5/6
LTBP	Latent TGF-β binding protein
MAML	Adaptor protein Mastermind-like
MAPK	Mitogen-activated protein kinase
M-CSF	Macrophage- colony stimulating factor
MH1	Mad homology 1 domain
miRNAs	microRNAs
MITF	Micropthalmia-associated transcription factor
MMP-9	Matrix metalloproteases
MSCs	Mesenchymal stem cells
NFATc1	Nuclear factor of activated T cells
NF-κB	Nuclear factor of κB
NICD	Notch intracellular domain
ODF	Osteoclast differentiation factor
OPG	Osteoprotegerin
OPGL	Osteoprotegerin ligand
OSCAR	Osteoclast-associated receptor
PI3K	Phosphoinositide 3-kinase
PKC	Protein kinase C
PLC	Phospholipase C
PPARγ	Peroxisome proliferation-activated receptor γ
PPM1A	Protein phosphatase magnesium-dependent 1A
RANKL	Receptor activator of nuclear factor kappa beta ligand
rhBMP2/7	rhBMP-2/BMP-7 heterodimer
R-Smad	Receptor-regulated Smad proteins
Runx2	Runt-related transcription factor 2
SARA	Smad anchor for receptor activation protein
sFRPs	Fz-related proteins
SGFs	Sarcoma growth factors
SIBLING	Small integrin-binding ligand N-linked glycoprotein
Smad	Small mothers against decapentaplegic
Smurf	Smad ubiquitin regulatory factor
TAB1	TAK1-binding protein 1
TACE	Tumor Necrosis Factor α-converting enzyme
TAK1	Transforming growth factor β-activated kinase 1
TβRII	TGF-βRII
TCF	T cell factor
TNF	Tumor necrosis factor
TGF-β	Transforming growth factor β
TRAFs	Tumor necrosis factor receptor-associated factors
TRANCE	Tumor necrosis factor-related activation-induced cytokine
TRAP	Tartrate-resistant acid phosphatase
TREM2	Triggering receptor expressed on myeloid cells-2
TSP1	Thrombospondin 1
v-ATPase	Vacuolar protons -transporting adenosine triphosphatase pump
VEGF	Vascular endothelial growth factor
